# Exposure to Agent Orange and Hepatocellular Carcinoma Among US Military Personnel

**DOI:** 10.1001/jamanetworkopen.2023.46380

**Published:** 2023-12-04

**Authors:** Jihane N. Benhammou, Mei Leng, Shailja C. Shah, George Cholankeril, Tien S. Dong, Arpan A. Patel, Myron J. Tong

**Affiliations:** 1 Greater Los Angeles Healthcare System, Los Angeles, California; 2 Department of Medicine, David Geffen School of Medicine, University of California, Los Angeles; 3 Jonsson Comprehensive Cancer Center, University of California, Los Angeles; 4 Biomathematics Department, University of California, Los Angeles; 5 VA San Diego Healthcare System, La Jolla, California; 6 Division of Gastroenterology, University of California, San Diego, La Jolla; 7 Baylor College of Medicine, Houston, Texas; 8 Huntington Medical Research Institutes, Pasadena, California

## Abstract

**Importance:**

Hepatocellular carcinoma (HCC) and its mortality are on the rise. Viral hepatitis and alcohol are leading risk factors; however, other risk factors among veterans are less defined, including Agent Orange (AO), an herbicide linked to several cancers.

**Objective:**

To assess the association of AO exposure and HCC in a national cohort of Vietnam veterans.

**Design, Setting, and Participants:**

This retrospective cohort study included Vietnam veterans who served between 1966 and 1975, were male, were older than 18 years at the time of deployment, and had established follow-up in the Veterans Affairs (VA) between 2000 and 2019. Veterans with AO exposure were identified in the disability data via validated clinical surveys. Relevant clinical risk factors for cirrhosis and HCC were collected. Patients were stratified based on cirrhosis status, as defined by consecutive diagnosis found by documented *International Classification of Diseases, Ninth Revision* and *International Statistical Classification of Diseases and Related Health Problems, Tenth Revision* scores or calculated Fibrosis-4 scores. Data were collected from January 1, 2019, to December 31, 2020, and analyzed from December 2020 to October 2023.

**Main Outcome and Measures:**

Incident HCC was the primary outcome. AO and HCC association was estimated using a multivariable Cox regression analysis, with death and liver transplant as competing events.

**Results:**

Of the 296 505 eligible veterans (222 545 [75.1%] White individuals and 44 342 [15.0%] Black individuals), 170 090 (57%) had AO exposure (mean [SD] age, 21.62 [3.49] years; 131 552 White individuals [83.2%] and 22 767 Black individuals [14.4%]) and 35 877 (12.1%) had cirrhosis. Veterans who were not exposed to AO were more likely to smoke (109 689 of 126 413 [86.8%] vs 146 061 of 170 090 [85.9%]); use alcohol (54 147 of 126 413 [42.8%] vs 71 951 of 170 090 [42.3%]) and have viral hepatitis (47 722 of 126 413 [37.8%] vs 58 942 of 170 090 [34.7%]). In a multivariable competing risk model, AO exposure was not associated with HCC. Among veterans with cirrhosis, self-identification as Hispanic individuals (aHR, 1.51; 95% CI, 1.30-1.75; *P* <.001) or Black individuals (aHR, 1.18; 95% CI, 1.05-1.32; *P* = .004), and having a diagnosis of viral hepatitis (aHR, 3.71; 95% CI, 3.26-4.24; *P* <.001), alcohol-associated liver disease (aHR, 1.32; 95% CI, 1.19-1.46; *P* <.001), and nonalcoholic fatty liver disease (NAFLD) (aHR, 1.92; 95% CI, 1.72-2.15; *P* <.001) were associated with HCC. Among veterans without cirrhosis, hypertension (aHR, 1.63; 95% CI, 1.23-2.15; *P* <.001) and diabetes (aHR, 1.52; 95% CI, 1.13-2.05; *P* = .005) were also associated with HCC. Early smoking and alcohol use were significant risk factors for HCC.

**Conclusions and Relevance:**

In this large nationwide cohort study of Vietnam veterans, AO exposure was not associated with HCC. Smoking, alcohol, viral hepatitis, and NAFLD were the most important clinical risk factors for HCC.

## Key Points

**Question:**

Is Agent Orange (AO) exposure during the Vietnam War (1966 to 1975) associated with incident hepatocellular carcinoma (HCC)?

**Findings:**

This cohort study of 296 505 eligible veterans, no association between AO and incident HCC was observed. However, among veterans, viral hepatitis, nonalcoholic fatty liver disease, and alcohol and tobacco use were the most important clinical risk factors for HCC, which were further modified by cirrhosis status.

**Meaning:**

These findings suggest that AO exposure is not associated with incident HCC.

## Teaser

This cohort study assessed the association of exposure to Agent Orange and hepatocellular carcinoma among Vietnam veterans.

## Introduction

Hepatocellular carcinoma (HCC) incidence is increasing globally with a relative 5-year survival of only 18%, with cirrhosis being the leading risk factor.^1^ Identifying key clinical drivers for HCC can guide screening practices of at-risk populations, which are associated with improved survivals^2^; can guide implementation of early interventions to modify risk factors; and has the potential to affect health care policy and delivery. For instance, as data emerged demonstrating the efficacy of mitigating the risk of liver complications with direct acting antiviral therapies for hepatitis C virus (HCV),^3^ stakeholders including the US Centers for Disease Control and Prevention and the US Preventative Services Task Force recommended universal HCV screening for adults.^4^ Similarly, as the metabolic syndrome and its hepatic manifestation of nonalcoholic fatty liver disease (NAFLD) were recognized an independent risk factors for HCC, society guidelines including recommended screening for NAFLD in patients with diabetes.^5^

The Veterans Healthcare Administration (VHA) is the largest integrated health care system in the United States serving 9 million veterans yearly. The VHA has placed a large emphasis on population health and implementing quality improvement measures to expedite the delivery of care to veterans. For instance, HCV screening and treatment were implemented in the VHA in a more comprehensive manner than any other health care system in the US.^6^ More recently, the national Hepatic Innovation Team Collaborative shifted their focus on improving the care of veterans living with cirrhosis by using automated and integrated clinical systems.^7,8^ Similarly, demonstrating how environmental exposures during combat affect health has also been emphasized within the VHA and is linked to a veterans’ medical service connection and ability to access health care.^9^ This is reflected in current government policies, including the 2022 Sergeant First Class Health Robinson Honoring Our Promise to Address Comprehensive Toxics (PACT) Act,^10^ signed into law by the Biden administration. The PACT Act is the largest health care benefit expansion for veteran exposed to toxins. This includes recognizing Agent Orange (AO)’s association with many malignant neoplasms.^11,12^

During the Vietnam war, the US military sprayed herbicides to defoliate forests to clear land of military occupation and control enemy food supplies.^13^ Most herbicides contained 2,4,5-trichlorophenoxyacetic acid (2,4,5-T), which was contaminated with 2,3,7,8-tetrachlorodibenzo-*p*-dioxin (TCDD), including in AO.^13^ It was recognized as early as 1970 that 2,4,5-T was teratogenic and was banned from US domestic use.^14^ An estimated 366 kg of TCDD was sprayed in South Vietnam,^15^ with large and persistent clinical ramifications to this day. Over the years, AO has been studied extensively given its association with several cancers, including bladder, Hodgkin and non-Hodgkin lymphoma, prostate, and lung and soft tissue sarcomas, leading to its classification in 1997 as a carcinogen by the International Agency for Research on Cancer.^16^ Its effects on HCC remain controversial. While animal models link TCDD to HCC,^17^ epidemiological studies in veterans have not been conclusive. These studies have been hindered by small or restricted sample sizes, short-term follow up, and a lack of consideration of other key clinical confounders for HCC, such as HCV and alcohol use, which are prevalent in veterans.^18,19,20,21,22^ Type 2 diabetes has also been linked to AO exposure and is recognized by the VHA as an AO-associated condition, which is also an independent risk factor for HCC.^23^ Given the new government incentives to expand eligibility of veterans and the lack of strong data linking AO to HCC, large, statistically rigorous studies addressing the limitations of previous work are needed to better evaluate if AO as a clinically relevant risk for HCC. In the current study, we investigate the association between AO and incident HCC using the largest cohort to-date of Vietnam veterans with a long-follow up in the VHA.

## Methods

This cohort study was conducted according to the Strengthening the Reporting of Observational Studies in Epidemiology (STROBE) reporting guideline.^24^ This study was approved by the institutional review board of the Veterans Affairs Greater Los Angeles Healthcare System (GLAHCS) with a waiver of informed consent because of the retrospective design.

### Data Source

The US VHA is comprised of 168 Veteran Affairs (VA) Medical Centers across the US and its territories and is the largest integrated, nationwide health care system in the US. The VHA contains an integrated network of electronic health data that can be accessed by approved researchers. For this study, we used the VA Informatics and Computer Infrastructure (VINCI) platform to extract demographics, clinical laboratory tests, medical comorbidities, alcohol use questionnaires and clinical outcomes data from the Corporate Data Warehouse (CDW), which is the national data repository for VA electronic health records.

### Study Cohort

To capture veterans who primarily obtained their care within the VHA, we selected veterans who had at least 2 outpatient visits that were separated by at least 6 months within a window of 3 years. From this group, we identified Vietnam veterans using their combat service location with the combat start date between the years January 1, 1966, and December 31, 1975. We excluded women (more than 99% of veterans deployed to Vietnam were males) and those who were younger than age 18 years at the time of deployment. Veterans were then followed up with from 2000 to 2019. The year 2000 was selected as the starting window because October 1999 was the earliest time when CDW data became available (eFigure in Supplement 1).

### Variable Definitions

#### AO Exposure

Veterans who were flagged as having AO exposure during the Vietnam War were categorized as AO exposed, while veterans deployed to Vietnam without exposure to AO were categorized as AO nonexposed. AO exposure was defined as any veteran who was flagged in the disability data under *AgentOrangeExposure*. These data are validated in the disability database to determine a veteran’s claim, which are defined clinically using the PACT Act.^10,25^ AO exposure is based on (1) having a health condition associated with AO per VHA (updated over time) and (2) having served in a location with exposure to AO. If a veteran believes they have a condition associated with AO but is not currently recognized by the VHA, the veteran must show evidence that the medical condition started during military service or scientific evidence (ie, publications) that show the condition is associated with AO. Additionally, we confirmed these methods to identify veterans who were AO exposed with VINCI Services. We also conducted a manual record review of a random set of 19 patients categorized as AO exposed who were seen at the local VA and found that 95% were correctly identified to have been exposed to AO during the Vietnam War.

#### Baseline Patient Characteristics

Relevant clinical covariates and baseline characteristics were selected a priori based on their association with HCC. Age at deployment, sex, race and ethnicity (self-reported by patients in electronic health records of the VHA) and medical comorbidities were extracted (eTable 1 in Supplement 1).^26,27^ All medical comorbidities were identified the first time they occurred in the veteran’s electronic health record. Diagnoses were identified using previously validated *International Classification of Diseases, Ninth Revision *(*ICD-9*) and *International Statistical Classification of Diseases and Related Health Problems, Tenth Revision *(*ICD-10*) codes.^26^ HIV and hepatitis B virus (HBV)^28^ or HCV^29^ were identified using laboratory data (HIV antibodies, HBV surface antigen, HCV RNA, respectively) (eTable 2 in Supplement 1). Alcohol-associated liver disease was defined as having an Alcohol Use Disorders Identification Test score of 4 or more for men^30^ or previously validated *ICD-9* and *ICD-10 *codes^27^ (eTable 1 in Supplement 1). Obesity was defined by a body mass index (BMI; calculated as weight in kilograms divided by height in meters squared) of more than 30, which was calculated from height (limited height by 48 to 84 inches) and weight (limited weight by 75 to 500 lbs) with the peak BMI recorded for each veteran.^31^ Smoking status was defined as nonsmoker (never or current nonsmoker in Health Factor table) or smoker (current smoker, former smoker, history of tobacco use).

#### Cirrhosis Ascertainment

A diagnosis of cirrhosis was defined using: (1) validated *ICD-9 *and *ICD-10* codes for cirrhosis^26^; (2) validated *ICD-9 *and *ICD-10* for decompensated cirrhosis (as defined by ascites, hepatic encephalopathy, hepatopulmonary syndrome, hepatorenal syndrome, spontaneous bacterial peritonitis, and variceal hemorrhage)^32^; or (3) having a Fibrosis-4 (FIB-4) score of more than 3.25 for at least 2 consecutive years (eTable 2 in Supplement 1).^33^ FIB-4 is calculated as follows using the aspartate aminotransferase (AST) level and alanine aminotransferase (ALT) value:

(Age [years] × AST level)/(Platelet count × √[ALT value])

All laboratory data in the FIB-4 calculations were within the last 3 months for each year. For each diagnosis, the earliest date was used to define the occurrence. While FIB-4 is a commonly used noninvasive tests clinically to assess the risk of cirrhosis, it can overestimate it given the large component of age, which can be a confounder given the aging population of Vietnam veterans.^34^ We next evaluated the number of patients with the validated *ICD-9 *and *ICD-10* for cirrhosis and decompensated cirrhosis and an elevated AST to Platelet Ratio Index (APRI) score^35^ (excludes age):

(AST level/AST upper limit of normal) / (Platelet count × 100)

### Outcomes

#### Primary Outcomes

HCC cases were identified using 2 methods. First, we used the VA Central Cancer Registry (VACCR), which constitutes a national repository of all cancer cases within the VHA since 1995. In the VACCR, each case is validated by a manual record abstraction using the North American Association for Central Cancer Registries standards with more than 90% accuracy.^36^ Second, we used *ICD-9 *and *ICD-10*^32^ codes if they occurred twice in the patient’s outpatient medical records, which has been well validated with positive estimated values of 84% to 94%.^33^ To assess the accuracy of the VACCR, we identified 40 cases and confirmed the diagnosis by conducting a manual record abstraction at GLAHCS. Of the 40 patients selected in the VACCR at GLAHCS, 90% were correctly identified, as confirmed by manual record abstraction of hepatology, gastroenterology, or oncology clinical notes. Using the *ICD-9 *and *ICD-10* codes, we identified 3469 veterans with HCC. Of those, we explored 31 cases, and HCC was correctly identified in 74% of the cases. We found that most incorrectly identified cases were secondary malignant neoplasms to the liver from other primary cancers, mostly of gastrointestinal origin (67% of the cases). To enrich cases with HCC using the *ICD-9 *and *ICD-10* algorithm, we identified all gastrointestinal cancer malignant neoplasms using *ICD-9 *and *ICD-10* (eTable 3 in Supplement 1) and removed them from the HCCs.

#### Secondary Outcomes

To identify death outcomes, we linked the VA CDW cohort to the VA-Department of Defense Mortality Data Repository (MDR),^37^ which provides reliable and quality-controlled data for death in veterans and a sensitivity of more than 90%.^38^ Next, we cross-referenced the death date from the VACCR, CDW, and MDR. We defined death outcomes as in MDR death file, in CDW death (if the date was between January 1, 2000, and December 31, 2019), or in VACCR death data. All death dates were between January 1, 2000, and December 31, 2019, with the latter date used as a censor date. Liver transplantation (LT) was identified using validated *ICD-9 *and *ICD-10* codes (eTable 1 in Supplement 1).^32,39^

### Statistical Analysis

Patient baseline characteristics are presented as the number and percentage of patients or mean (SD). Covariates were selected based on known clinical associations with HCC. HBV and HCV were combined into 1 covariate (viral hepatitis). NASH and NAFLD diagnoses were also combined into 1 covariate. Diabetes, hypertension, and HBV and HCV were used as time-dependent covariates. Given the link between diabetes and obesity, we assessed the interaction between the 2 diagnoses. Interactions were also assessed between AO and viral hepatitis, age, alcohol, NAFLD or NASH, smoking, and diabetes.

We conducted a competing risk analysis to assess the risk of incident HCC as a primary outcome with death and LT as competing events. Type-specific adjusted hazard ratios (aHRs) are reported for both univariate and multivariate models. Covariate selection was determined a priori and included age (at deployment); race and ethnicity; smoking; portal hypertension; and etiologies of cirrhosis, including NAFLD or NASH, viral hepatitis, alcohol associated liver disease, hemochromatosis, alpha-1-antitrypsin, secondary biliary or unspecified biliary cirrhosis, and autoimmune hepatitis. Given that cirrhosis is the most important risk factor for HCC and that cirrhosis could not be ascertained until after CDW were available, which was approximately in the year 2000, the results were stratified into cirrhosis and noncirrhosis status for each veteran prior to any statistical analyses. Furthermore, to adjust for the impact of cirrhosis on HCC, we conducted inverse probability weighting (IPW) model.

All statistical analyses were conducted using SAS version 9.4 (SAS Institute). Statistical significance was set at *P* <.05, and tests were 2-sided. Data were collected from January 1, 2019, to December 31, 2020, and analyzed from December 2020 to October 2023.

## Results

### Study Population

We identified 296 505 male veterans during the Vietnam War who were 18 years or older at the time of deployment (222 545 White individuals [75.1%] and 44 342 Black individuals [15.0%]). Of these veterans, 170 090 (57%) had AO exposure (mean [SD] age, 21.62 [3.49] years; 131 552 White individuals [83.2%] and 22 767 Black individuals [14.4%]) (Table 1). ALD was the most prevalent cause of chronic liver disease (126 098 [42.5%]), followed by viral hepatitis (HBV and HCV) (106 664 [36.0%]), and NAFLD or NASH (36 761 [12.4%]). The metabolic syndrome was also prevalent with 160 190 patients (54.0%) being diagnosed with diabetes, 260 484 (87.9%) with dyslipidemia, and 258 973 (87.3%) with hypertension. While statistically significant because of the large sample size, there did not appear to be a clinical difference in active smoking status (109 689 of 126 413 [86.8%] vs 146 061 of 170 090 [85.8%]; *P* <.001) nor alcohol use (54 147 of 126 413 [42.8%] vs 71 951 of 170 090 [42.3%]; *P* = .004) among veterans based on AO exposure (Table 1). Cirrhosis was more commonly found in patients who were not exposed to AO, compared with patients who were exposed to AO (16 611 of 126 413 [13.1%] vs 19 266 of 170 090 [11.3%]; *P* <.001). Most veterans who were exposed to AO had been deployed for 1 year or less (175 796 veterans; [60.9%]; cumulative percent, 60.9%), 91 014 veterans (31.5%; cumulative percent, 92.4%) had been deployed for 1 to 2 years, 13 510 veterans (4.7%; cumulative percent, 97.1%) had been deployed 2 to 3 years, 5031 veterans (1.7%; cumulative percent, 98.8%) had been deployed 3 to 4 years, and 3428 veterans (1.2%; cumulative percent, 100%) had been deployed 4 or more years. Mean (SD) deployment time was 1.11 (0.66) years.

**Table 1.** Patient Characteristics

Characteristic	Patients, No. (%)		*P* value
	AO exposed (n = 170 090)	AO not exposed (n = 126 413)	
Age, mean (SD), y	21.62 (3.49)	21.94 (4.06)	<.001
Race			
American Indian or Alaskan Native	1688 (1.1)	1153 (1.0)	<.001
Asian	648 (0.4)	724 (0.6)	
Black or African American	22 767 (14.4)	21 575 (18.7)	
Native Hawaiian or Pacific Islander	1461 (0.9)	1198 (1.0)	
White	131 552 (83.2)	90 993 (78.7)	
Ethnicity			
Hispanic or Latino	9503 (5.8)	6040 (5.0)	<.001
Nonliver-related comorbidities			
Smoking	146 061 (85.8)	109 689 (86.8)	<.001
Obesity	110 025 (64.7)	81 334 (64.4)	.06
Type 2 diabetes	91 421 (53.8)	68 769 (54.4)	<.001
Hypertension	148 309 (87.2)	110 664 (87.5)	.005
Dyslipidemia	149 976 (88.2)	110 508 (87.4)	<.001
HIV	46 310 (27.2)	37 062 (29.3)	<.001
Liver-related comorbidities			
Ethanol use or alcohol liver disease	71 951 (42.3)	54 147 (42.8)	.004
Virus hepatitis (HBV, HCV)	58 942 (34.7)	47 722 (37.8)	<.001
NAFLD/NASH	20914 (12.3)	15 847 (12.5)	.05
Hemochromatosis	1726 (1.01)	1279 (1.01)	.96
Alpha-1-antitrypsin	1946 (1.14)	1568 (1.2)	.02
Autoimmune liver disease	924 (0.5)	750 (0.6)	.07
Secondary or unspecific biliary cirrhosis	166 (0.1)	140 (0.1)	.27
Wilson disease	48 (0.03)	36 (0.03)	.97
Cirrhosis	19 266 (11.3)	16 611 (13.1)	<.001

Abbreviations: AO, agent orange; HBV, hepatitis B virus; HCV, hepatitis C virus; NAFLD, nonalcoholic fatty liver disease; NASH, nonalcoholic steatohepatitis.

### AO Exposure and HCC Risk

#### Incident HCC

At a mean (SD) of follow-up time is 51.1 (1.9) years (51.2 [1.8] years and 51.0 [2.1] years in AO exposed and AO nonexposed group, respectively), we found 2654 veterans who developed HCC in our final cohort (1463 [0.9%] in the AO exposed group and 1191 [0.9%] in the AO nonexposed group). HCC incidence rate was 0.00018 per year (0.00018 per year and 0.00017 per year). In our multivariable model adjusting for other confounders of HCC, we found that AO was not associated with HCC both in veterans with cirrhosis (aHR, 1.00; 95% CI, 0.91-1.10; *P* = .98) and without cirrhosis (aHR, 1.05; 95% CI, 0.89-1.23; *P* = .57). The lack of association between AO and incident HCC based on cirrhosis status was also substantiated using the APRI score, which is another surrogate marker for advanced fibrosis and cirrhosis (eTable 4 in Supplement 1). Next, we conducted IPW to adjust for cirrhosis and did not find a significant association between AO exposure and incident HCC (eTable 5 in Supplement 1).

To further confirm our findings and explore the association of death data accuracy, we conducted a sensitivity analysis using (1) HCC as an event with death as the only competing risk, and (2) HCC as an event and death was the censored event if occurred before HCC (instead of censored at last follow up in 2019) and transplantation as the competing risk. After adjusting for all the HCC risk factors, neither analysis found an association between AO and HCC risk. Given the inverse association between age and incident HCC, we explored the association between AO and age further. There were no significant interactions between age and AO exposure which was further confirmed by assessing their association after stratification of age at deployment (eMethods in Supplement 1).

#### LT or Death

There was no difference in LT occurrence based on AO exposure status (366 of 170 090 [0.22%] vs 296 of 126 413 [0.23%]). Overall mortality was lower in the AO exposed (12 589 of 23 706 [7.4%]) vs nonexposed group (11 117 of 23 706 [8.8%]).

### Other Risk Factors Associated With HCC

We found that self-reported race and ethnicity were associated with incident HCC (Table 2; eTable 4 in Supplement 1). Veterans with cirrhosis who self-identified as Hispanic or Latino individuals (aHR, 1.51; 95% CI, 1.30-1.75; *P* <.001) or Black individuals (aHR, 1.18; 95% CI, 1.05-1.32; *P* = .004) had a higher risk for HCC compared with non-Hispanic White individuals.

**Table 2.** Univariable and Multivariable Models for HCC Development With Liver Transplantation and Death as Competing Events

Characteristic	Noncirrhosis (n = 260 628)				Cirrhosis (n = 35 877)^a^			
	Univariable, HR (95% CI)^b^	*P* value	Multivariable, aHR (95% CI)	*P* value	Univariable, HR (95% CI)^b^	*P* value	Multivariable, aHR (95% CI)	*P* value
Agent Orange	0.95 (0.81-1.11)	.52	NA	NA	0.98 (0.92-1.09)	.98	NA	NA
Age (at deployment)	0.99 (0.97-1.01)	.31	1.02 (1.00-1.04)	.04	0.89 (0.87-0.91)	<.001	0.96 (0.95-0.98)	<.001
Race and ethnicity								
Non-Hispanic Black	1.50 (1.22-1.83)	<.001	NA	NA	1.40 (1.26-1.56)	<.001	1.18 (1.05-1.32)	.004
Hispanic or Latino	1.21 (0.88-1.66)	.25	NA	NA	1.93 (1.67-2.22)	<.001	1.51 (1.30-1.75)	<.001
Non-Hispanic White	1 [Reference]	NA	1 [Reference]	NA	1 [Reference]	NA	1 [Reference]	NA
Smoking	1.64 (1.25-2.16)	<.001	NA	NA	1.27 (1.05-1.53)	.015	NA	NA
Obesity	1.25 (1.06-1.48)	.009	NA	NA	0.89 (0.81-0.98)	.013	NA	NA
Viral hepatitis	3.10 (2.64-3.64)	<.001	2.42 (2.04-2.87)	<.001	5.68 (5.00-6.46)	<.001	3.71 (3.26-4.24)	<.001
Alcohol liver disease	1.27 (1.05-1.43)	.001	NA	NA	2.04 (1.84-2.25)	<.001	1.32 (1.19-1.46)	<.001
NASH/NAFLD^c^	5.77 (4.88-6.81)	<.001	2.39 (1.92-2.98)	<.001	4.28 (3.89-4.70)	<.001	1.92 (1.72-2.15)	<.001
HIV	1.37 (1.16-1.61)	<.001	NA	NA	1.28 (1.17-1.40)	<.001	NA	NA
Autoimmune hepatitis^d^	5.40 (2.97-9.83)	<.001	NA	NA	2.20 (1.81-2.68)	<.001	NA	NA
Hemochromatosis	2.72 (1.63-4.54)	<.001	1.73 (1.03-2.91)	.04	2.12 (1.74-2.59)	<.001	1.32 (1.08-1.63)	.008
Alpha-1-antitrypsin	2.68 (1.57-4.55)	<.001	1.74 (1.02-2.98)	.04	1.69 (1.42-2.01)	<.001	NA	NA
Secondary or unspecific biliary cirrhosis^e^	NA	NA	NA	NA	3.37 (2.54-4.47)	<.001	NA	NA
Dyslipidemia	0.72 (0.58-0.90)	.004	0.46 (0.36-0.58)	<.001	0.37 (0.33-0.41)	<.001	0.46 (0.41-0.51)	<.001
Hypertension	2.15 (1.64-2.82)	<.001	1.63 (1.23-2.15)	<.001	1.11 (0.95-1.29)	.20	NA	NA
Diabetes^f^	1.73 (1.47-2.05)	<.001	1.52 (1.13-2.05)	.005	1.02 (0.93-1.12)	.65	NA	NA

Abbreviations: aHR, adjusted hazard ratio; HBV, hepatitis B virus; HCV, hepatitis C virus; HR, hazard ratio; NA, not applicable; NAFLD, nonalcoholic fatty liver disease; NASH, nonalcoholic steatohepatitis.

^a^ Cirrhosis defined using validated *International Classification of Diseases, Ninth Revision* and *International Statistical Classification of Diseases and Related Health Problems, Tenth Revision* and consecutive Fibrosis-4 scores.

^b^ Adjusting for age at deployment and race and ethnicity.

^c^ Combined NAFLD and NASH in multivariable model.

^d^ Combined HCV and HBV.

^e^ No cases in the noncirrhosis group.

^f^ Obesity and diabetes interactions assessed which was not significant in multivariable model.

In the subgroup of patients with cirrhosis (35 877 [12.1%]), the most important risk factors for HCC included viral hepatitis (aHR, 3.71; 95% CI, 3.26-4.24; *P *<.001), NAFLD or NASH (aHR, 1.92; 95% CI, 1.72-2.15; *P* <.001), and ALD (aHR, 1.32; 95% CI, 1.19-1.46; *P* <.001). Dyslipidemia was inversely associated with the risk of HCC (aHR, 0.46; 95% CI, 0.41-0.51; *P* <.001) in this cohort. Diabetes (aHR, 1.50; 95% CI, 1.13-2.01; *P* = .008) and hypertension (aHR, 1.63; 95% CI, 1.23-2.15; *P* <.001) were only significant risk factors in the noncirrhosis group, even after adjusting for obesity and assessing the interactions between obesity and type 2 diabetes.

Age at deployment was inversely associated with incident HCC in veterans with cirrhosis (aHR, 0.96; 95% CI 0.95-0.98; *P* <.001). To explore this further, we assessed the association between smoking and alcohol with age for veterans with and without cirrhosis and stratified age by decades. For both alcohol and smoking, we found that early exposure during ages 20 to 30 years were significantly associated with incident HCC, independent of cirrhosis status (eTables 6 to 8 in Supplement 1). While we found an association between incident HCC and age at deployment in veterans without cirrhosis (aHR, 1.02; 95% CI, 1.00-1.04; *P* = .047), this was not reproduced using the age-independent APRI score (eTable 4 in Supplement 1).

## Discussion

In this large nationwide analysis of approximately 300 000 Vietnam veterans, we report on key, modifiable clinical risk factors associated with incident HCC. After adjusting for relevant clinical risk factors in a competing risk model, we found that AO exposure was not associated with incident HCC.

HCC is a complex disease that results from many clinical and environmental risk factors that can change over time.^40^ AO has been linked diabetes,^11^ which is independently associated with incident HCC.^23,32^ We explored these associations in our analyses but did not find that AO exposure modified the HCC risk. While our study aimed to investigate the association of AO with HCC, we found other important associations. Dyslipidemia was inversely associated with the risk of HCC in veterans irrespective of cirrhosis and after adjusting for confounders. Although we did not assess the association of statin use, we hypothesize that the inverse association between dyslipidemia and incident HCC is driven by statins because they have been reported to decrease HCC incidence and improve overall survival in patients with cirrhosis.^41,42,43^ Early exposure to alcohol and tobacco use were also important risks for HCC development, especially in veterans with cirrhosis. Taken together, our work supports that HCC risk factors are dynamic and can change during the course of a veteran’s life depending on the presence of cirrhosis and other contributing factors, such as alcohol and tobacco use. These findings, in conjunction with our novel findings that AO is not associated with HCC, help inform which risk factors (ie, viral hepatitis, NAFLD or NASH, and ALD) should be prioritized and modified to mitigate the rise in HCC among veterans.

To appropriately assess the risk of AO exposure and HCC development, we defined our control group as veterans who were deployed to Vietnam but were not flagged in the VHA as having been exposed to AO. This study design allows to control for other nonmeasurable factors, such as other potential geographical, diet, and environmental-related exposures that are not captured by the electronic health records. Using a non-Vietnam War group as a control may not have appropriately isolated these differences and could have potentially introduced bias. This is especially relevant given the consideration of the healthy soldier effect (HSE) that has previously been described, where veterans who are deployed are less likely to develop chronic diseases, including during the Vietnam War.^44^ The HSE has been attributed to early physical training and health care access and has been shown to occur as far out as 40 years from war.^45^

We found that self-reported race and ethnicity were significantly associated with incident HCC. Specifically, patients who self-identified as Hispanic or non-Hispanic Black were more likely to develop HCC, compared with White individuals, even after adjusting for key clinical confounders. These findings suggest that other nonmeasured variables could contribute to these disparities. Several studies have described that Black individuals are at higher risk for HCC-related death, independently of tumor stage.^46,47,48,49,50,51^ However, few studies have addressed this in the VHA. Our findings support that even within the VHA, where patients have access to health care, racial and ethnic disparities persist. Small studies have proposed that mental health and substance use may drive these disparities; however, larger studies are needed to understand these observations.

We found that a minority of veterans underwent LT. While LT is a life-saving measure for patients with advanced liver disease,^52^ it remains limited resource due to the scarcity of organs and a complex evaluation process that affects candidates every level of the evaluation, including referral, eligibility and listing, especially in veterans who have been shown to disproportionally receive fewer LTs.^53^ Therefore, HCC risk modification of clinical factors is even more imperative in veterans to prevent HCC.

### Limitations

While we present the largest cohort of US Vietnam veterans with AO exposure with long-term follow, our study has notable limitations. Due to the retrospective nature of the study, residual confounders between exposure and outcomes may be present. While we used robust methods to identify AO exposure, veterans who may not claim an AO-associated disability may not have been captured accordingly. Others have used similar methods to ours to identify AO exposed cases; however, we recognize that since AO cannot be directly measured, there is a potential for misclassification of cases. Using the age at deployment allowed us to determine if there was a potential dose-response given the length of exposure; however, this was not identified. The long follow-up period of our cohort allowed us to identify HCC cases, which can take 20 to 40 years^54^ to occur and thus may not be captured in shorter studies. However, given the limitation of data availability within the VHA only starting in the years 1999 and 2000, events prior these dates would not have been available for our review. Therefore, our cohort may be biased to a potentially healthier veteran population. Although sex differences have been described in HCC, this could not be addressed in our cohort given that less than 1% of all Vietnam veterans were female. Rare etiologies of liver disease, such as Wilson disease (84 veterans), could not be robustly evaluated in our study. Finally, since our interest was to capture veterans who primarily received their care within the VHA, we could not capture events that occurred outside of the VHA, including for those associated with liver transplantation.

## Conclusions

Our results suggest that AO was not a significant risk factor for HCC development and does not appear to modify the association between other known risk factors, such as diabetes, on HCC risk. We show that HCC risk is dynamic based on whether or not there is a background of cirrhosis and that risk modification should be tailored to the veteran’s individual risk.

## Notes

Supplement 1. **eFigure.** Flow Diagram of Vietnam Veteran Cohort **eTable 1.** List of ICD-9 and ICD-10 Codes Used for Etiologies of HCC and Medical Comorbidities **eTable 2.** List of Laboratory LOINCs Including HIV, HCV, HBV, ALT, AST and Platelet Counts **eTable 3.** ICD-9 Codes of Gastrointestinal Malignant Neoplasms Other Than HCC **eTable 4.** Association Between AO and Other Risk Factors for HCC Using APRI Score to Identify HCC Cases **eMethods.** Additional Methods With eTables 5-8 **eTable 5.** Association Between AO and Other Risk Factors for HCC Using Inverse Probability Weighing **eTable 6.** Age (In Years) Stratification for AO Exposure **eTable 7.** Age (In Years) Stratification for Tobacco Use **eTable 8. **Age (In Years) Stratification for Alcohol Use

Supplement 2.
Data Sharing Statement
