# Supplementary material for: Exposure to Agent Orange and Hepatocellular Carcinoma Among US Military Personnel
Source: JAMA Netw Open. 2023 Dec 4;6(12):e2346380. doi: 10.1001/jamanetworkopen.2023.46380 (PMC10696483; doi:10.1001/jamanetworkopen.2023.46380)
Supplement: Supplement 1. — eFigure. Flow Diagram of Vietnam Veteran Cohort eTable 1. List of ICD-9 and ICD-10 Codes Used for Etiologies of HCC and Medical Comorbidities eTable 2. List of Laboratory LOINCs Including HIV, HCV, HBV, ALT, AST and Platelet Counts eTable 3. ICD-9 Codes of Gastrointestinal Malignant Neoplasms Other Than HCC eTable 4. Association Between AO and Other Risk Factors for HCC Using APRI Score to Identify HCC Cases eMethods. Additional Methods With eTables 5-8 eTable 5. Association Between AO and Other Risk Factors for HCC Using Inverse Probability Weighing eTable 6. Age (In Years) Stratification for AO Exposure eTable 7. Age (In Years) Stratification for Tobacco Use eTable 8. Age (In Years) Stratification for Alcohol Use [file jamanetwopen-e2346380-s001.pdf]

Supplemental Online Content

Benhammou JN, Leng M, Shah, SC, et al. Exposure to Agent Orange and hepatocellular carcinoma among US military personnel. *JAMA Netw Open*. 2023;6(12):e2346380. doi:10.1001/jamanetworkopen.2023.46380

eFigure. Flow Diagram of Vietnam Veteran Cohort
eTable 1. List of *ICD-9* and *ICD-10* Codes Used for Etiologies of HCC and Medical Comorbidities
eTable 2. List of Laboratory LOINCs Including HIV, HCV, HBV, ALT, AST and Platelet Counts
eTable 3. *ICD-9* Codes of Gastrointestinal Malignant Neoplasms Other Than HCC
eTable 4. Association Between AO and Other Risk Factors for HCC Using APRI Score to Identify HCC Cases
eMethods. Additional Methods With eTables 5-8
eTable 5. Association Between AO and Other Risk Factors for HCC Using Inverse Probability Weighing
eTable 6. Age (In Years) Stratification for AO Exposure
eTable 7. Age (In Years) Stratification for Tobacco Use
eTable 8. Age (In Years) Stratification for Alcohol Use

This supplemental material has been provided by the authors to give readers additional information about their work.

© 2023 Benhammou JN et al. *JAMA Network Open.*

eFigure 1- Flow diagram of final cohort

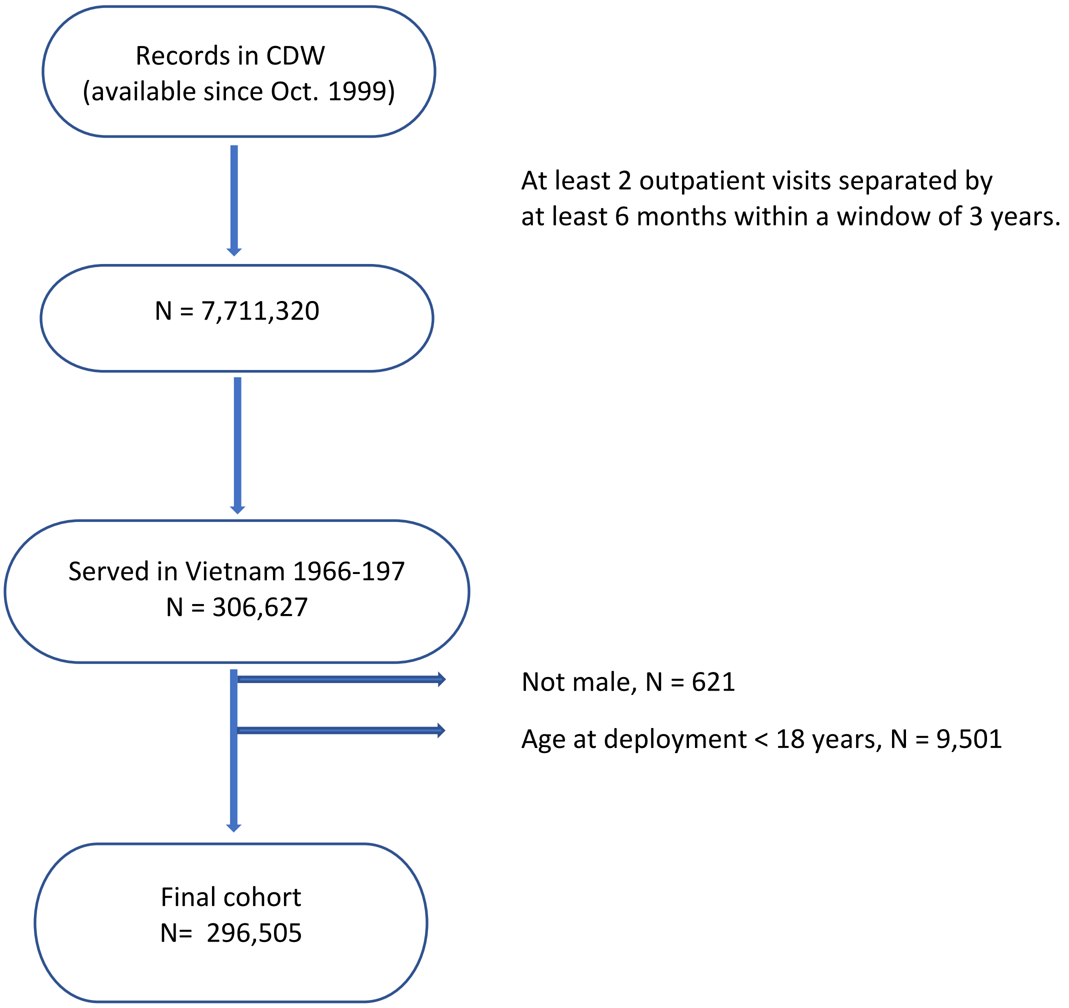

© 2023 Benhammou JN et al. *JAMA Network Open.*

eTable 1. List of *ICD-9* and *ICD-10* Codes Used for Etiologies of HCC and Medical Comorbidities

eTable 1a- viral hepatitis ICD 9/10 codes

ICD 9/10	CONCEPT_NAME
70	Viral hepatitis
70.1	Viral hepatitis A without mention of hepatic coma
70.2	Viral hepatitis B with hepatic coma
70.2	Viral hepatitis B with hepatic coma, acute or unspecified, without mention of hepatitis delta
70.21	Viral hepatitis B with hepatic coma, acute or unspecified, with hepatitis delta
70.22	Chronic viral hepatitis B with hepatic coma without hepatitis delta
70.23	Chronic viral hepatitis B with hepatic coma with hepatitis delta
70.3	Viral hepatitis B without mention of hepatic coma
70.3	Viral hepatitis B without mention of hepatic coma, acute or unspecified, without mention of hepatitis delta
70.31	Viral hepatitis B without mention of hepatic coma, acute or unspecified, with hepatitis delta
70.32	Chronic viral hepatitis B without mention of hepatic coma without mention of hepatitis delta
70.32	Chronic viral hepatitis B without mention of hepatic coma without mention of hepatitis delta
70.33	Chronic viral hepatitis B without mention of hepatic coma with hepatitis delta
70.33	Chronic viral hepatitis B without mention of hepatic coma with hepatitis delta
70.4	Other specified viral hepatitis with hepatic coma
70.41	Acute hepatitis C with hepatic coma
70.42	Hepatitis delta without mention of active hepatitis B disease with hepatic coma
70.42	Hepatitis delta without mention of active hepatitis B disease with hepatic coma
70.44	Chronic hepatitis C with hepatic coma
70.49	Other specified viral hepatitis with hepatic coma
70.5	Other specified viral hepatitis without mention of hepatic coma
70.51	Acute hepatitis C without mention of hepatic coma
70.52	Hepatitis delta without mention of active hepatitis B disease or hepatic coma
70.54	Chronic hepatitis C without mention of hepatic coma
70.59	Other specified viral hepatitis without mention of hepatic coma
70.6	Unspecified viral hepatitis with hepatic coma
70.7	Unspecified viral hepatitis C
70.7	Unspecified viral hepatitis C without hepatic coma
70.71	Unspecified viral hepatitis C with hepatic coma
70.9	Unspecified viral hepatitis without mention of hepatic coma
B16	Acute hepatitis B
B16.0	Acute hepatitis B with delta-agent with hepatic coma
B16.1	Acute hepatitis B with delta-agent without hepatic coma
B16.2	Acute hepatitis B without delta-agent with hepatic coma
B16.9	Acute hepatitis B without delta-agent and without hepatic coma
B17	Other acute viral hepatitis
B17.0	Acute delta-(super) infection of hepatitis B carrier
B17.1	Acute hepatitis C
B17.10	Acute hepatitis C without hepatic coma
B17.11	Acute hepatitis C with hepatic coma
B17.8	Other specified acute viral hepatitis
B17.9	Acute viral hepatitis, unspecified
B18	Chronic viral hepatitis

© 2023 Benhammou JN et al. *JAMA Network Open.*

B18.0	Chronic viral hepatitis B with delta-agent
B18.1	Chronic viral hepatitis B without delta-agent
B18.2	Chronic viral hepatitis C
B18.8	Other chronic viral hepatitis
B18.9	Chronic viral hepatitis, unspecified
B19	Unspecified viral hepatitis
B19.0	Unspecified viral hepatitis with hepatic coma
B19.1	Unspecified viral hepatitis B
B19.10	Unspecified viral hepatitis B without hepatic coma
B19.11	Unspecified viral hepatitis B with hepatic coma
B19.2	Unspecified viral hepatitis C
B19.20	Unspecified viral hepatitis C without hepatic coma
B19.21	Unspecified viral hepatitis C with hepatic coma
B19.9	Unspecified viral hepatitis without hepatic coma

eTable 1b- ICD 9/10 codes for alcohol associated liver disease (ALD)

ICD 9/10	CONCEPT_NAME
291	Alcohol-induced mental disorders
291.0	Alcohol withdrawal delirium
291.1	Alcohol-induced persisting amnestic disorder
291.2	Alcohol-induced persisting dementia
291.3	Alcohol-induced psychotic disorder with hallucinations
291.4	Idiosyncratic alcohol intoxication
291.5	Alcohol-induced psychotic disorder with delusions
291.8	Other specified alcohol-induced mental disorders
291.81	Alcohol withdrawal
291.82	Alcohol induced sleep disorders
291.89	Other alcohol-induced mental disorders
291.9	Unspecified alcohol-induced mental disorders
303	Alcohol dependence syndrome
303.0	Acute alcoholic intoxication
303.00	Acute alcoholic intoxication in alcoholism, unspecified
303.01	Acute alcoholic intoxication in alcoholism, continuous
303.02	Acute alcoholic intoxication in alcoholism, episodic
303.03	Acute alcoholic intoxication in alcoholism, in remission
303.9	Other and unspecified alcohol dependence
303.90	Other and unspecified alcohol dependence, unspecified
303.91	Other and unspecified alcohol dependence, continuous
303.92	Other and unspecified alcohol dependence, episodic
303.93	Other and unspecified alcohol dependence, in remission
305.0	Alcohol abuse
305.00	Alcohol abuse, unspecified
305.01	Alcohol abuse, continuous
305.02	Alcohol abuse, episodic
305.03	Alcohol abuse, in remission
357.5	Alcoholic polyneuropathy

© 2023 Benhammou JN et al. *JAMA Network Open.*

425.5	Alcoholic cardiomyopathy
535.3	Alcoholic gastritis
535.3	Alcoholic gastritis, without mention of hemorrhage
535.31	Alcoholic gastritis, with hemorrhage
571.0	Alcoholic fatty liver
571.1	Acute alcoholic hepatitis
571.2	Alcoholic cirrhosis of liver
571.3	Alcoholic liver damage, unspecified
790.3	Excessive blood level of alcohol
F10	Alcohol related disorders
F10.1	Alcohol abuse
F10.10	Alcohol abuse, uncomplicated
F10.11	Alcohol abuse, in remission
F10.12	Alcohol abuse with intoxication
F10.120	Alcohol abuse with intoxication, uncomplicated
F10.121	Alcohol abuse with intoxication delirium
F10.129	Alcohol abuse with intoxication, unspecified
F10.13	Alcohol abuse, with withdrawal
F10.130	Alcohol abuse with withdrawal, uncomplicated
F10.131	Alcohol abuse with withdrawal delirium
F10.132	Alcohol abuse with withdrawal with perceptual disturbance
F10.139	Alcohol abuse with withdrawal, unspecified
F10.14	Alcohol abuse with alcohol-induced mood disorder
F10.15	Alcohol abuse with alcohol-induced psychotic disorder
F10.150	Alcohol abuse with alcohol-induced psychotic disorder with delusions
F10.151	Alcohol abuse with alcohol-induced psychotic disorder with hallucinations
F10.159	Alcohol abuse with alcohol-induced psychotic disorder, unspecified
F10.18	Alcohol abuse with other alcohol-induced disorders
F10.180	Alcohol abuse with alcohol-induced anxiety disorder
F10.181	Alcohol abuse with alcohol-induced sexual dysfunction
F10.182	Alcohol abuse with alcohol-induced sleep disorder
F10.188	Alcohol abuse with other alcohol-induced disorder
F10.19	Alcohol abuse with unspecified alcohol-induced disorder
F10.2	Alcohol dependence
F10.20	Alcohol dependence, uncomplicated
F10.21	Alcohol dependence, in remission
F10.22	Alcohol dependence with intoxication
F10.220	Alcohol dependence with intoxication, uncomplicated
F10.221	Alcohol dependence with intoxication delirium
F10.229	Alcohol dependence with intoxication, unspecified
F10.23	Alcohol dependence with withdrawal
F10.230	Alcohol dependence with withdrawal, uncomplicated
F10.231	Alcohol dependence with withdrawal delirium

© 2023 Benhammou JN et al. *JAMA Network Open.*

F10.232	Alcohol dependence with withdrawal with perceptual disturbance
F10.239	Alcohol dependence with withdrawal, unspecified
F10.24	Alcohol dependence with alcohol-induced mood disorder
F10.25	Alcohol dependence with alcohol-induced psychotic disorder
F10.250	Alcohol dependence with alcohol-induced psychotic disorder with delusions
F10.251	Alcohol dependence with alcohol-induced psychotic disorder with hallucinations
F10.259	Alcohol dependence with alcohol-induced psychotic disorder, unspecified
F10.26	Alcohol dependence with alcohol-induced persisting amnestic disorder
F10.27	Alcohol dependence with alcohol-induced persisting dementia
F10.28	Alcohol dependence with other alcohol-induced disorders
F10.280	Alcohol dependence with alcohol-induced anxiety disorder
F10.281	Alcohol dependence with alcohol-induced sexual dysfunction
F10.282	Alcohol dependence with alcohol-induced sleep disorder
F10.288	Alcohol dependence with other alcohol-induced disorder
F10.29	Alcohol dependence with unspecified alcohol-induced disorder
F10.9	Alcohol use, unspecified
F10.92	Alcohol use, unspecified with intoxication
F10.920	Alcohol use, unspecified with intoxication, uncomplicated
F10.921	Alcohol use, unspecified with intoxication delirium
F10.929	Alcohol use, unspecified with intoxication, unspecified
F10.93	Alcohol use, unspecified with withdrawal
F10.930	Alcohol use, unspecified with withdrawal, uncomplicated
F10.931	Alcohol use, unspecified with withdrawal delirium
F10.932	Alcohol use, unspecified with withdrawal with perceptual disturbance
F10.939	Alcohol use, unspecified with withdrawal, unspecified
F10.94	Alcohol use, unspecified with alcohol-induced mood disorder
F10.95	Alcohol use, unspecified with alcohol-induced psychotic disorder
F10.950	Alcohol use, unspecified with alcohol-induced psychotic disorder with delusions
F10.951	Alcohol use, unspecified with alcohol-induced psychotic disorder with hallucinations
F10.959	Alcohol use, unspecified with alcohol-induced psychotic disorder, unspecified
F10.96	Alcohol use, unspecified with alcohol-induced persisting amnestic disorder
F10.97	Alcohol use, unspecified with alcohol-induced persisting dementia
F10.98	Alcohol use, unspecified with other alcohol-induced disorders
F10.980	Alcohol use, unspecified with alcohol-induced anxiety disorder
F10.981	Alcohol use, unspecified with alcohol-induced sexual dysfunction
F10.982	Alcohol use, unspecified with alcohol-induced sleep disorder

© 2023 Benhammou JN et al. *JAMA Network Open.*

F10.988	Alcohol use, unspecified with other alcohol-induced disorder
F10.99	Alcohol use, unspecified with unspecified alcohol-induced disorder
G31.2	Degeneration of nervous system due to alcohol
G62.1	Alcoholic polyneuropathy
G72.1	Alcoholic myopathy
I42.6	Alcoholic cardiomyopathy
K29.2	Alcoholic gastritis
K29.20	Alcoholic gastritis without bleeding
K29.21	Alcoholic gastritis with bleeding
K70	Alcoholic liver disease
K70.0	Alcoholic fatty liver
K70.1	Alcoholic hepatitis
K70.10	Alcoholic hepatitis without ascites
K70.11	Alcoholic hepatitis with ascites
K70.2	Alcoholic fibrosis and sclerosis of liver
K70.3	Alcoholic cirrhosis of liver
K70.30	Alcoholic cirrhosis of liver without ascites
K70.31	Alcoholic cirrhosis of liver with ascites
K70.4	Alcoholic hepatic failure
K70.40	Alcoholic hepatic failure without coma
K70.41	Alcoholic hepatic failure with coma
K70.9	Alcoholic liver disease, unspecified
Z71.4	Alcohol abuse counseling and surveillance
Z71.41	Alcohol abuse counseling and surveillance of alcoholic

eTable 1c- Nonalcoholic fatty liver disease (NAFLD) and nonalcoholic steatohepatitis (NASH) ICD 9/10 codes.

ICD 9/10	CONCEPT_NAME
571.8	chronic nonalcoholic steatohepatiits
571.9	unspecified chronic liver disease
573.8	liver disorders
573.9	unspecified disorder of liver
K75.8	other specified inflammatory liver diseases
571.8	chronic nonalcoholic liver disease
K76.0	fatty (change of) liver

eTable 1d- Hemochromatosis ICD 9/10codes

ICD 9/10	CONCEPT_NAME
275	Disorders of mineral metabolism
E83.1	Disorders of iron metabaolism

eTable 1e- autoimmune liver disease ICD 9/10 codes

ICD 9/10	CONCEPT_NAME
571.6	Biliary cirrhosis

© 2023 Benhammou JN et al. *JAMA Network Open.*

576.1	Cholangitis
K74.3	Primary biliary cirrhosis
K75.4	Autoimmune hepatitis
K83.0	Cholangitis
K83.01	Primary sclerosing cholangitis
K83.09	Other cholangitis

eTable 1f- ICD 9/10 codes for other liver diseases

ICD9/10	CONCEPT_NAME
572.8	Liver disease sequelae
576.8	Biliary cyst
277.09	Cystic fibrosis and othe manifestations
K76.89	Other specified diseases of the liver
K83.8	Other specified diseases of biliary tract
E84.8	Cystic fibrosis and othe manifestations
K74.0	Hepatic fibrosis

eTable 1g- Alpha-1-anti trypsin ICD 9/10 codes

ICD 9/10	CONCEPT_NAME
277.6	Other deficiencies of circulating enzymes
E88.0	Disorders of plasma-protein metabolism, not elsewhere classified
E88.02	Plasminogen deficiency
E88.01	Alpha-1-antitrypsin deficiency
E88.09	Other disorders of plasma-protein metabolism, not elsewhere classified

eTable 1h- Diabetes ICD 9/10 codes

ICD 9/10	CONCEPT_NAME
E08.0	Diabetes mellitus due to underlying condition with hyperosmolarity
E08.00	Diabetes mellitus due to underlying condition with hyperosmolarity without nonketotic hyperglycemic-hyperosmolar coma (NKHHC)
E08.01	Diabetes mellitus due to underlying condition with hyperosmolarity with coma
E08.1	Diabetes mellitus due to underlying condition with ketoacidosis
E08.10	Diabetes mellitus due to underlying condition with ketoacidosis without coma
E08.11	Diabetes mellitus due to underlying condition with ketoacidosis with coma
E08.2	Diabetes mellitus due to underlying condition with kidney complications
E08.21	Diabetes mellitus due to underlying condition with diabetic nephropathy
E08.22	Diabetes mellitus due to underlying condition with diabetic chronic kidney disease
E08.29	Diabetes mellitus due to underlying condition with other diabetic kidney complication
E08.3	Diabetes mellitus due to underlying condition with ophthalmic complications
E08.31	Diabetes mellitus due to underlying condition with unspecified diabetic retinopathy
E08.311	Diabetes mellitus due to underlying condition with unspecified diabetic retinopathy with macular edema
E08.319	Diabetes mellitus due to underlying condition with unspecified diabetic retinopathy without macular edema
E08.32	Diabetes mellitus due to underlying condition with mild nonproliferative diabetic retinopathy

© 2023 Benhammou JN et al. *JAMA Network Open.*

E08.321	Diabetes mellitus due to underlying condition with mild nonproliferative diabetic retinopathy with macular edema
E08.3211	Diabetes mellitus due to underlying condition with mild nonproliferative diabetic retinopathy with macular edema, right eye
E08.3212	Diabetes mellitus due to underlying condition with mild nonproliferative diabetic retinopathy with macular edema, left eye
E08.3213	Diabetes mellitus due to underlying condition with mild nonproliferative diabetic retinopathy with macular edema, bilateral
E08.3219	Diabetes mellitus due to underlying condition with mild nonproliferative diabetic retinopathy with macular edema, unspecified eye
E08.329	Diabetes mellitus due to underlying condition with mild nonproliferative diabetic retinopathy without macular edema
E08.3291	Diabetes mellitus due to underlying condition with mild nonproliferative diabetic retinopathy without macular edema, right eye
E08.3292	Diabetes mellitus due to underlying condition with mild nonproliferative diabetic retinopathy without macular edema, left eye
E08.3293	Diabetes mellitus due to underlying condition with mild nonproliferative diabetic retinopathy without macular edema, bilateral
E08.3299	Diabetes mellitus due to underlying condition with mild nonproliferative diabetic retinopathy without macular edema, unspecified eye
E08.33	Diabetes mellitus due to underlying condition with moderate nonproliferative diabetic retinopathy
E08.331	Diabetes mellitus due to underlying condition with moderate nonproliferative diabetic retinopathy with macular edema
E08.3311	Diabetes mellitus due to underlying condition with moderate nonproliferative diabetic retinopathy with macular edema, right eye
E08.3312	Diabetes mellitus due to underlying condition with moderate nonproliferative diabetic retinopathy with macular edema, left eye
E08.3313	Diabetes mellitus due to underlying condition with moderate nonproliferative diabetic retinopathy with macular edema, bilateral
E08.3319	Diabetes mellitus due to underlying condition with moderate nonproliferative diabetic retinopathy with macular edema, unspecified eye
E08.339	Diabetes mellitus due to underlying condition with moderate nonproliferative diabetic retinopathy without macular edema
E08.3391	Diabetes mellitus due to underlying condition with moderate nonproliferative diabetic retinopathy without macular edema, right eye
E08.3392	Diabetes mellitus due to underlying condition with moderate nonproliferative diabetic retinopathy without macular edema, left eye
E08.3393	Diabetes mellitus due to underlying condition with moderate nonproliferative diabetic retinopathy without macular edema, bilateral
E08.3399	Diabetes mellitus due to underlying condition with moderate nonproliferative diabetic retinopathy without macular edema, unspecified eye
E08.34	Diabetes mellitus due to underlying condition with severe nonproliferative diabetic retinopathy
E08.341	Diabetes mellitus due to underlying condition with severe nonproliferative diabetic retinopathy with macular edema
E08.3411	Diabetes mellitus due to underlying condition with severe nonproliferative diabetic retinopathy with macular edema, right eye
E08.3412	Diabetes mellitus due to underlying condition with severe nonproliferative diabetic retinopathy with macular edema, left eye
E08.3413	Diabetes mellitus due to underlying condition with severe nonproliferative diabetic retinopathy with macular edema, bilateral
E08.3419	Diabetes mellitus due to underlying condition with severe nonproliferative diabetic retinopathy with macular edema, unspecified eye

© 2023 Benhammou JN et al. *JAMA Network Open.*

E08.349	Diabetes mellitus due to underlying condition with severe nonproliferative diabetic retinopathy without macular edema
E08.3491	Diabetes mellitus due to underlying condition with severe nonproliferative diabetic retinopathy without macular edema, right eye
E08.3492	Diabetes mellitus due to underlying condition with severe nonproliferative diabetic retinopathy without macular edema, left eye
E08.3493	Diabetes mellitus due to underlying condition with severe nonproliferative diabetic retinopathy without macular edema, bilateral
E08.3499	Diabetes mellitus due to underlying condition with severe nonproliferative diabetic retinopathy without macular edema, unspecified eye
E08.35	Diabetes mellitus due to underlying condition with proliferative diabetic retinopathy
E08.351	Diabetes mellitus due to underlying condition with proliferative diabetic retinopathy with macular edema
E08.3511	Diabetes mellitus due to underlying condition with proliferative diabetic retinopathy with macular edema, right eye
E08.3512	Diabetes mellitus due to underlying condition with proliferative diabetic retinopathy with macular edema, left eye
E08.3513	Diabetes mellitus due to underlying condition with proliferative diabetic retinopathy with macular edema, bilateral
E08.3519	Diabetes mellitus due to underlying condition with proliferative diabetic retinopathy with macular edema, unspecified eye
E08.352	Diabetes mellitus due to underlying condition with proliferative diabetic retinopathy with traction retinal detachment involving the macula
E08.3521	Diabetes mellitus due to underlying condition with proliferative diabetic retinopathy with traction retinal detachment involving the macula, right eye
E08.3522	Diabetes mellitus due to underlying condition with proliferative diabetic retinopathy with traction retinal detachment involving the macula, left eye
E08.3523	Diabetes mellitus due to underlying condition with proliferative diabetic retinopathy with traction retinal detachment involving the macula, bilateral
E08.3529	Diabetes mellitus due to underlying condition with proliferative diabetic retinopathy with traction retinal detachment involving the macula, unspecified eye
E08.353	Diabetes mellitus due to underlying condition with proliferative diabetic retinopathy with traction retinal detachment not involving the macula
E08.3531	Diabetes mellitus due to underlying condition with proliferative diabetic retinopathy with traction retinal detachment not involving the macula, right eye
E08.3532	Diabetes mellitus due to underlying condition with proliferative diabetic retinopathy with traction retinal detachment not involving the macula, left eye
E08.3533	Diabetes mellitus due to underlying condition with proliferative diabetic retinopathy with traction retinal detachment not involving the macula, bilateral
E08.3539	Diabetes mellitus due to underlying condition with proliferative diabetic retinopathy with traction retinal detachment not involving the macula, unspecified eye
E08.354	Diabetes mellitus due to underlying condition with proliferative diabetic retinopathy with combined traction retinal detachment and rhegmatogenous retinal detachment
E08.3541	Diabetes mellitus due to underlying condition with proliferative diabetic retinopathy with combined traction retinal detachment and rhegmatogenous retinal detachment, right eye
E08.3542	Diabetes mellitus due to underlying condition with proliferative diabetic retinopathy with combined traction retinal detachment and rhegmatogenous retinal detachment, left eye
E08.3543	Diabetes mellitus due to underlying condition with proliferative diabetic retinopathy with combined traction retinal detachment and rhegmatogenous retinal detachment, bilateral
E08.3549	Diabetes mellitus due to underlying condition with proliferative diabetic retinopathy with combined traction retinal detachment and rhegmatogenous retinal detachment, unspecified eye
E08.355	Diabetes mellitus due to underlying condition with stable proliferative diabetic retinopathy

© 2023 Benhammou JN et al. *JAMA Network Open.*

E08.3551	Diabetes mellitus due to underlying condition with stable proliferative diabetic retinopathy, right eye
E08.3552	Diabetes mellitus due to underlying condition with stable proliferative diabetic retinopathy, left eye
E08.3553	Diabetes mellitus due to underlying condition with stable proliferative diabetic retinopathy, bilateral
E08.3559	Diabetes mellitus due to underlying condition with stable proliferative diabetic retinopathy, unspecified eye
E08.359	Diabetes mellitus due to underlying condition with proliferative diabetic retinopathy without macular edema
E08.3591	Diabetes mellitus due to underlying condition with proliferative diabetic retinopathy without macular edema, right eye
E08.3592	Diabetes mellitus due to underlying condition with proliferative diabetic retinopathy without macular edema, left eye
E08.3593	Diabetes mellitus due to underlying condition with proliferative diabetic retinopathy without macular edema, bilateral
E08.3599	Diabetes mellitus due to underlying condition with proliferative diabetic retinopathy without macular edema, unspecified eye
E08.36	Diabetes mellitus due to underlying condition with diabetic cataract
E08.37	Diabetes mellitus due to underlying condition with diabetic macular edema, resolved following treatment
E08.37X1	Diabetes mellitus due to underlying condition with diabetic macular edema, resolved following treatment, right eye
E08.37X2	Diabetes mellitus due to underlying condition with diabetic macular edema, resolved following treatment, left eye
E08.37X3	Diabetes mellitus due to underlying condition with diabetic macular edema, resolved following treatment, bilateral
E08.37X9	Diabetes mellitus due to underlying condition with diabetic macular edema, resolved following treatment, unspecified eye
E08.39	Diabetes mellitus due to underlying condition with other diabetic ophthalmic complication
E08.4	Diabetes mellitus due to underlying condition with neurological complications
E08.40	Diabetes mellitus due to underlying condition with diabetic neuropathy, unspecified
E08.41	Diabetes mellitus due to underlying condition with diabetic mononeuropathy
E08.42	Diabetes mellitus due to underlying condition with diabetic polyneuropathy
E08.43	Diabetes mellitus due to underlying condition with diabetic autonomic (poly)neuropathy
E08.44	Diabetes mellitus due to underlying condition with diabetic amyotrophy
E08.49	Diabetes mellitus due to underlying condition with other diabetic neurological complication
E08.5	Diabetes mellitus due to underlying condition with circulatory complications
E08.51	Diabetes mellitus due to underlying condition with diabetic peripheral angiopathy without gangrene
E08.52	Diabetes mellitus due to underlying condition with diabetic peripheral angiopathy with gangrene
E08.59	Diabetes mellitus due to underlying condition with other circulatory complications
E08.6	Diabetes mellitus due to underlying condition with other specified complications
E08.61	Diabetes mellitus due to underlying condition with diabetic arthropathy
E08.610	Diabetes mellitus due to underlying condition with diabetic neuropathic arthropathy
E08.618	Diabetes mellitus due to underlying condition with other diabetic arthropathy
E08.62	Diabetes mellitus due to underlying condition with skin complications
E08.620	Diabetes mellitus due to underlying condition with diabetic dermatitis
E08.621	Diabetes mellitus due to underlying condition with foot ulcer
E08.622	Diabetes mellitus due to underlying condition with other skin ulcer
E08.628	Diabetes mellitus due to underlying condition with other skin complications

© 2023 Benhammou JN et al. *JAMA Network Open.*

E08.63	Diabetes mellitus due to underlying condition with oral complications
E08.630	Diabetes mellitus due to underlying condition with periodontal disease
E08.638	Diabetes mellitus due to underlying condition with other oral complications
E08.64	Diabetes mellitus due to underlying condition with hypoglycemia
E08.640	Diabetes mellitus due to underlying condition with hypoglycemia without coma
E08.641	Diabetes mellitus due to underlying condition with hypoglycemia with coma
E08.649	Diabetes mellitus due to underlying condition with hypoglycemia without coma
E08.65	Diabetes mellitus due to underlying condition with hyperglycemia
E08.69	Diabetes mellitus due to underlying condition with other specified complication
E08.8	Diabetes mellitus due to underlying condition with unspecified complications
E08.9	Diabetes mellitus due to underlying condition without complications
E09.0	Drug or chemical induced diabetes mellitus with hyperosmolarity
E09.00	Drug or chemical induced diabetes mellitus with hyperosmolarity without nonketotic hyperglycemic-hyperosmolar coma (NKHHC)
E09.01	Drug or chemical induced diabetes mellitus with hyperosmolarity with coma
E09.1	Drug or chemical induced diabetes mellitus with ketoacidosis
E09.10	Drug or chemical induced diabetes mellitus with ketoacidosis without coma
E09.11	Drug or chemical induced diabetes mellitus with ketoacidosis with coma
E09.2	Drug or chemical induced diabetes mellitus with kidney complications
E09.21	Drug or chemical induced diabetes mellitus with diabetic nephropathy
E09.22	Drug or chemical induced diabetes mellitus with diabetic chronic kidney disease
E09.29	Drug or chemical induced diabetes mellitus with other diabetic kidney complication
E09.3	Drug or chemical induced diabetes mellitus with ophthalmic complications
E09.31	Drug or chemical induced diabetes mellitus with unspecified diabetic retinopathy
E09.311	Drug or chemical induced diabetes mellitus with unspecified diabetic retinopathy with macular edema
E09.319	Drug or chemical induced diabetes mellitus with unspecified diabetic retinopathy without macular edema
E09.32	Drug or chemical induced diabetes mellitus with mild nonproliferative diabetic retinopathy
E09.321	Drug or chemical induced diabetes mellitus with mild nonproliferative diabetic retinopathy with macular edema
E09.3211	Drug or chemical induced diabetes mellitus with mild nonproliferative diabetic retinopathy with macular edema, right eye
E09.3212	Drug or chemical induced diabetes mellitus with mild nonproliferative diabetic retinopathy with macular edema, left eye
E09.3213	Drug or chemical induced diabetes mellitus with mild nonproliferative diabetic retinopathy with macular edema, bilateral
E09.3219	Drug or chemical induced diabetes mellitus with mild nonproliferative diabetic retinopathy with macular edema, unspecified eye
E09.329	Drug or chemical induced diabetes mellitus with mild nonproliferative diabetic retinopathy without macular edema
E09.3291	Drug or chemical induced diabetes mellitus with mild nonproliferative diabetic retinopathy without macular edema, right eye
E09.3292	Drug or chemical induced diabetes mellitus with mild nonproliferative diabetic retinopathy without macular edema, left eye
E09.3293	Drug or chemical induced diabetes mellitus with mild nonproliferative diabetic retinopathy without macular edema, bilateral
E09.3299	Drug or chemical induced diabetes mellitus with mild nonproliferative diabetic retinopathy without macular edema, unspecified eye
E09.33	Drug or chemical induced diabetes mellitus with moderate nonproliferative diabetic retinopathy

© 2023 Benhammou JN et al. *JAMA Network Open.*

E09.331	Drug or chemical induced diabetes mellitus with moderate nonproliferative diabetic retinopathy with macular edema
E09.3311	Drug or chemical induced diabetes mellitus with moderate nonproliferative diabetic retinopathy with macular edema, right eye
E09.3312	Drug or chemical induced diabetes mellitus with moderate nonproliferative diabetic retinopathy with macular edema, left eye
E09.3313	Drug or chemical induced diabetes mellitus with moderate nonproliferative diabetic retinopathy with macular edema, bilateral
E09.3319	Drug or chemical induced diabetes mellitus with moderate nonproliferative diabetic retinopathy with macular edema, unspecified eye
E09.339	Drug or chemical induced diabetes mellitus with moderate nonproliferative diabetic retinopathy without macular edema
E09.3391	Drug or chemical induced diabetes mellitus with moderate nonproliferative diabetic retinopathy without macular edema, right eye
E09.3392	Drug or chemical induced diabetes mellitus with moderate nonproliferative diabetic retinopathy without macular edema, left eye
E09.3393	Drug or chemical induced diabetes mellitus with moderate nonproliferative diabetic retinopathy without macular edema, bilateral
E09.3399	Drug or chemical induced diabetes mellitus with moderate nonproliferative diabetic retinopathy without macular edema, unspecified eye
E09.34	Drug or chemical induced diabetes mellitus with severe nonproliferative diabetic retinopathy
E09.341	Drug or chemical induced diabetes mellitus with severe nonproliferative diabetic retinopathy with macular edema
E09.3411	Drug or chemical induced diabetes mellitus with severe nonproliferative diabetic retinopathy with macular edema, right eye
E09.3412	Drug or chemical induced diabetes mellitus with severe nonproliferative diabetic retinopathy with macular edema, left eye
E09.3413	Drug or chemical induced diabetes mellitus with severe nonproliferative diabetic retinopathy with macular edema, bilateral
E09.3419	Drug or chemical induced diabetes mellitus with severe nonproliferative diabetic retinopathy with macular edema, unspecified eye
E09.349	Drug or chemical induced diabetes mellitus with severe nonproliferative diabetic retinopathy without macular edema
E09.3491	Drug or chemical induced diabetes mellitus with severe nonproliferative diabetic retinopathy without macular edema, right eye
E09.3492	Drug or chemical induced diabetes mellitus with severe nonproliferative diabetic retinopathy without macular edema, left eye
E09.3493	Drug or chemical induced diabetes mellitus with severe nonproliferative diabetic retinopathy without macular edema, bilateral
E09.3499	Drug or chemical induced diabetes mellitus with severe nonproliferative diabetic retinopathy without macular edema, unspecified eye
E09.35	Drug or chemical induced diabetes mellitus with proliferative diabetic retinopathy
E09.351	Drug or chemical induced diabetes mellitus with proliferative diabetic retinopathy with macular edema
E09.3511	Drug or chemical induced diabetes mellitus with proliferative diabetic retinopathy with macular edema, right eye
E09.3512	Drug or chemical induced diabetes mellitus with proliferative diabetic retinopathy with macular edema, left eye
E09.3513	Drug or chemical induced diabetes mellitus with proliferative diabetic retinopathy with macular edema, bilateral
E09.3519	Drug or chemical induced diabetes mellitus with proliferative diabetic retinopathy with macular edema, unspecified eye

© 2023 Benhammou JN et al. *JAMA Network Open.*

E09.352	Drug or chemical induced diabetes mellitus with proliferative diabetic retinopathy with traction retinal detachment involving the macula
E09.3521	Drug or chemical induced diabetes mellitus with proliferative diabetic retinopathy with traction retinal detachment involving the macula, right eye
E09.3522	Drug or chemical induced diabetes mellitus with proliferative diabetic retinopathy with traction retinal detachment involving the macula, left eye
E09.3523	Drug or chemical induced diabetes mellitus with proliferative diabetic retinopathy with traction retinal detachment involving the macula, bilateral
E09.3529	Drug or chemical induced diabetes mellitus with proliferative diabetic retinopathy with traction retinal detachment involving the macula, unspecified eye
E09.353	Drug or chemical induced diabetes mellitus with proliferative diabetic retinopathy with traction retinal detachment not involving the macula
E09.3531	Drug or chemical induced diabetes mellitus with proliferative diabetic retinopathy with traction retinal detachment not involving the macula, right eye
E09.3532	Drug or chemical induced diabetes mellitus with proliferative diabetic retinopathy with traction retinal detachment not involving the macula, left eye
E09.3533	Drug or chemical induced diabetes mellitus with proliferative diabetic retinopathy with traction retinal detachment not involving the macula, bilateral
E09.3539	Drug or chemical induced diabetes mellitus with proliferative diabetic retinopathy with traction retinal detachment not involving the macula, unspecified eye
E09.354	Drug or chemical induced diabetes mellitus with proliferative diabetic retinopathy with combined traction retinal detachment and rhegmatogenous retinal detachment
E09.3541	Drug or chemical induced diabetes mellitus with proliferative diabetic retinopathy with combined traction retinal detachment and rhegmatogenous retinal detachment, right eye
E09.3542	Drug or chemical induced diabetes mellitus with proliferative diabetic retinopathy with combined traction retinal detachment and rhegmatogenous retinal detachment, left eye
E09.3543	Drug or chemical induced diabetes mellitus with proliferative diabetic retinopathy with combined traction retinal detachment and rhegmatogenous retinal detachment, bilateral
E09.3549	Drug or chemical induced diabetes mellitus with proliferative diabetic retinopathy with combined traction retinal detachment and rhegmatogenous retinal detachment, unspecified eye
E09.355	Drug or chemical induced diabetes mellitus with stable proliferative diabetic retinopathy
E09.3551	Drug or chemical induced diabetes mellitus with stable proliferative diabetic retinopathy, right eye
E09.3552	Drug or chemical induced diabetes mellitus with stable proliferative diabetic retinopathy, left eye
E09.3553	Drug or chemical induced diabetes mellitus with stable proliferative diabetic retinopathy, bilateral
E09.3559	Drug or chemical induced diabetes mellitus with stable proliferative diabetic retinopathy, unspecified eye
E09.359	Drug or chemical induced diabetes mellitus with proliferative diabetic retinopathy without macular edema
E09.3591	Drug or chemical induced diabetes mellitus with proliferative diabetic retinopathy without macular edema, right eye
E09.3592	Drug or chemical induced diabetes mellitus with proliferative diabetic retinopathy without macular edema, left eye
E09.3593	Drug or chemical induced diabetes mellitus with proliferative diabetic retinopathy without macular edema, bilateral
E09.3599	Drug or chemical induced diabetes mellitus with proliferative diabetic retinopathy without macular edema, unspecified eye
E09.36	Drug or chemical induced diabetes mellitus with diabetic cataract
E09.37	Drug or chemical induced diabetes mellitus with diabetic macular edema, resolved following treatment
E09.37X1	Drug or chemical induced diabetes mellitus with diabetic macular edema, resolved following treatment, right eye

© 2023 Benhammou JN et al. *JAMA Network Open.*

E09.37X2	Drug or chemical induced diabetes mellitus with diabetic macular edema, resolved following treatment, left eye
E09.37X3	Drug or chemical induced diabetes mellitus with diabetic macular edema, resolved following treatment, bilateral
E09.37X9	Drug or chemical induced diabetes mellitus with diabetic macular edema, resolved following treatment, unspecified eye
E09.39	Drug or chemical induced diabetes mellitus with other diabetic ophthalmic complication
E09.4	Drug or chemical induced diabetes mellitus with neurological complications
E09.40	Drug or chemical induced diabetes mellitus with neurological complications with diabetic neuropathy, unspecified
E09.41	Drug or chemical induced diabetes mellitus with neurological complications with diabetic mononeuropathy
E09.42	Drug or chemical induced diabetes mellitus with neurological complications with diabetic polyneuropathy
E09.43	Drug or chemical induced diabetes mellitus with neurological complications with diabetic autonomic (poly)neuropathy
E09.44	Drug or chemical induced diabetes mellitus with neurological complications with diabetic amyotrophy
E09.49	Drug or chemical induced diabetes mellitus with neurological complications with other diabetic neurological complication
E09.5	Drug or chemical induced diabetes mellitus with circulatory complications
E09.51	Drug or chemical induced diabetes mellitus with diabetic peripheral angiopathy without gangrene
E09.52	Drug or chemical induced diabetes mellitus with diabetic peripheral angiopathy with gangrene
E09.59	Drug or chemical induced diabetes mellitus with other circulatory complications
E09.6	Drug or chemical induced diabetes mellitus with other specified complications
E09.61	Drug or chemical induced diabetes mellitus with diabetic arthropathy
E09.610	Drug or chemical induced diabetes mellitus with diabetic neuropathic arthropathy
E09.618	Drug or chemical induced diabetes mellitus with other diabetic arthropathy
E09.62	Drug or chemical induced diabetes mellitus with skin complications
E09.620	Drug or chemical induced diabetes mellitus with diabetic dermatitis
E09.621	Drug or chemical induced diabetes mellitus with foot ulcer
E09.622	Drug or chemical induced diabetes mellitus with other skin ulcer
E09.628	Drug or chemical induced diabetes mellitus with other skin complications
E09.63	Drug or chemical induced diabetes mellitus with oral complications
E09.630	Drug or chemical induced diabetes mellitus with periodontal disease
E09.638	Drug or chemical induced diabetes mellitus with other oral complications
E09.65	Drug or chemical induced diabetes mellitus with hyperglycemia
E09.69	Drug or chemical induced diabetes mellitus with other specified complication
E09.8	Drug or chemical induced diabetes mellitus with unspecified complications
E09.9	Drug or chemical induced diabetes mellitus without complications
E11	Type 2 diabetes mellitus
E11.0	Type 2 diabetes mellitus with hyperosmolarity
E11.00	Type 2 diabetes mellitus with hyperosmolarity without nonketotic hyperglycemic-hyperosmolar coma (NKHHC)
E11.01	Type 2 diabetes mellitus with hyperosmolarity with coma
E11.1	Type 2 diabetes mellitus with ketoacidosis
E11.10	Type 2 diabetes mellitus with ketoacidosis without coma
E11.11	Type 2 diabetes mellitus with ketoacidosis with coma
E11.2	Type 2 diabetes mellitus with kidney complications
E11.21	Type 2 diabetes mellitus with diabetic nephropathy

E11.22	Type 2 diabetes mellitus with diabetic chronic kidney disease
E11.29	Type 2 diabetes mellitus with other diabetic kidney complication
E11.3	Type 2 diabetes mellitus with ophthalmic complications
E11.31	Type 2 diabetes mellitus with unspecified diabetic retinopathy
E11.311	Type 2 diabetes mellitus with unspecified diabetic retinopathy with macular edema
E11.319	Type 2 diabetes mellitus with unspecified diabetic retinopathy without macular edema
E11.32	Type 2 diabetes mellitus with mild nonproliferative diabetic retinopathy
E11.321	Type 2 diabetes mellitus with mild nonproliferative diabetic retinopathy with macular edema
E11.3211	Type 2 diabetes mellitus with mild nonproliferative diabetic retinopathy with macular edema, right eye
E11.3212	Type 2 diabetes mellitus with mild nonproliferative diabetic retinopathy with macular edema, left eye
E11.3213	Type 2 diabetes mellitus with mild nonproliferative diabetic retinopathy with macular edema, bilateral
E11.3219	Type 2 diabetes mellitus with mild nonproliferative diabetic retinopathy with macular edema, unspecified eye
E11.329	Type 2 diabetes mellitus with mild nonproliferative diabetic retinopathy without macular edema
E11.3291	Type 2 diabetes mellitus with mild nonproliferative diabetic retinopathy without macular edema, right eye
E11.3292	Type 2 diabetes mellitus with mild nonproliferative diabetic retinopathy without macular edema, left eye
E11.3293	Type 2 diabetes mellitus with mild nonproliferative diabetic retinopathy without macular edema, bilateral
E11.3299	Type 2 diabetes mellitus with mild nonproliferative diabetic retinopathy without macular edema, unspecified eye
E11.33	Type 2 diabetes mellitus with moderate nonproliferative diabetic retinopathy
E11.331	Type 2 diabetes mellitus with moderate nonproliferative diabetic retinopathy with macular edema
E11.3311	Type 2 diabetes mellitus with moderate nonproliferative diabetic retinopathy with macular edema, right eye
E11.3312	Type 2 diabetes mellitus with moderate nonproliferative diabetic retinopathy with macular edema, left eye
E11.3313	Type 2 diabetes mellitus with moderate nonproliferative diabetic retinopathy with macular edema, bilateral
E11.3319	Type 2 diabetes mellitus with moderate nonproliferative diabetic retinopathy with macular edema, unspecified eye
E11.339	Type 2 diabetes mellitus with moderate nonproliferative diabetic retinopathy without macular edema
E11.3391	Type 2 diabetes mellitus with moderate nonproliferative diabetic retinopathy without macular edema, right eye
E11.3392	Type 2 diabetes mellitus with moderate nonproliferative diabetic retinopathy without macular edema, left eye
E11.3393	Type 2 diabetes mellitus with moderate nonproliferative diabetic retinopathy without macular edema, bilateral
E11.3399	Type 2 diabetes mellitus with moderate nonproliferative diabetic retinopathy without macular edema, unspecified eye
E11.34	Type 2 diabetes mellitus with severe nonproliferative diabetic retinopathy
E11.341	Type 2 diabetes mellitus with severe nonproliferative diabetic retinopathy with macular edema
E11.3411	Type 2 diabetes mellitus with severe nonproliferative diabetic retinopathy with macular edema, right eye
E11.3412	Type 2 diabetes mellitus with severe nonproliferative diabetic retinopathy with macular edema, left eye

© 2023 Benhammou JN et al. *JAMA Network Open.*

E11.3413	Type 2 diabetes mellitus with severe nonproliferative diabetic retinopathy with macular edema, bilateral
E11.3419	Type 2 diabetes mellitus with severe nonproliferative diabetic retinopathy with macular edema, unspecified eye
E11.349	Type 2 diabetes mellitus with severe nonproliferative diabetic retinopathy without macular edema
E11.3491	Type 2 diabetes mellitus with severe nonproliferative diabetic retinopathy without macular edema, right eye
E11.3492	Type 2 diabetes mellitus with severe nonproliferative diabetic retinopathy without macular edema, left eye
E11.3493	Type 2 diabetes mellitus with severe nonproliferative diabetic retinopathy without macular edema, bilateral
E11.3499	Type 2 diabetes mellitus with severe nonproliferative diabetic retinopathy without macular edema, unspecified eye
E11.35	Type 2 diabetes mellitus with proliferative diabetic retinopathy
E11.351	Type 2 diabetes mellitus with proliferative diabetic retinopathy with macular edema
E11.3511	Type 2 diabetes mellitus with proliferative diabetic retinopathy with macular edema, right eye
E11.3512	Type 2 diabetes mellitus with proliferative diabetic retinopathy with macular edema, left eye
E11.3513	Type 2 diabetes mellitus with proliferative diabetic retinopathy with macular edema, bilateral
E11.3519	Type 2 diabetes mellitus with proliferative diabetic retinopathy with macular edema, unspecified eye
E11.352	Type 2 diabetes mellitus with proliferative diabetic retinopathy with traction retinal detachment involving the macula
E11.3521	Type 2 diabetes mellitus with proliferative diabetic retinopathy with traction retinal detachment involving the macula, right eye
E11.3522	Type 2 diabetes mellitus with proliferative diabetic retinopathy with traction retinal detachment involving the macula, left eye
E11.3523	Type 2 diabetes mellitus with proliferative diabetic retinopathy with traction retinal detachment involving the macula, bilateral
E11.3529	Type 2 diabetes mellitus with proliferative diabetic retinopathy with traction retinal detachment involving the macula, unspecified eye
E11.353	Type 2 diabetes mellitus with proliferative diabetic retinopathy with traction retinal detachment not involving the macula
E11.3531	Type 2 diabetes mellitus with proliferative diabetic retinopathy with traction retinal detachment not involving the macula, right eye
E11.3532	Type 2 diabetes mellitus with proliferative diabetic retinopathy with traction retinal detachment not involving the macula, left eye
E11.3533	Type 2 diabetes mellitus with proliferative diabetic retinopathy with traction retinal detachment not involving the macula, bilateral
E11.3539	Type 2 diabetes mellitus with proliferative diabetic retinopathy with traction retinal detachment not involving the macula, unspecified eye
E11.354	Type 2 diabetes mellitus with proliferative diabetic retinopathy with combined traction retinal detachment and rhegmatogenous retinal detachment
E11.3541	Type 2 diabetes mellitus with proliferative diabetic retinopathy with combined traction retinal detachment and rhegmatogenous retinal detachment, right eye
E11.3542	Type 2 diabetes mellitus with proliferative diabetic retinopathy with combined traction retinal detachment and rhegmatogenous retinal detachment, left eye
E11.3543	Type 2 diabetes mellitus with proliferative diabetic retinopathy with combined traction retinal detachment and rhegmatogenous retinal detachment, bilateral

© 2023 Benhammou JN et al. *JAMA Network Open.*

E11.354 9	Type 2 diabetes mellitus with proliferative diabetic retinopathy with combined traction retinal detachment and rhegmatogenous retinal detachment, unspecified eye
E11.355	Type 2 diabetes mellitus with stable proliferative diabetic retinopathy
E11.355 1	Type 2 diabetes mellitus with stable proliferative diabetic retinopathy, right eye
E11.355 2	Type 2 diabetes mellitus with stable proliferative diabetic retinopathy, left eye
E11.355 3	Type 2 diabetes mellitus with stable proliferative diabetic retinopathy, bilateral
E11.355 9	Type 2 diabetes mellitus with stable proliferative diabetic retinopathy, unspecified eye
E11.359	Type 2 diabetes mellitus with proliferative diabetic retinopathy without macular edema
E11.359 1	Type 2 diabetes mellitus with proliferative diabetic retinopathy without macular edema, right eye
E11.359 2	Type 2 diabetes mellitus with proliferative diabetic retinopathy without macular edema, left eye
E11.359 3	Type 2 diabetes mellitus with proliferative diabetic retinopathy without macular edema, bilateral
E11.359 9	Type 2 diabetes mellitus with proliferative diabetic retinopathy without macular edema, unspecified eye
E11.36	Type 2 diabetes mellitus with diabetic cataract
E11.37	Type 2 diabetes mellitus with diabetic macular edema, resolved following treatment
E11.37X 1	Type 2 diabetes mellitus with diabetic macular edema, resolved following treatment, right eye
E11.37X 2	Type 2 diabetes mellitus with diabetic macular edema, resolved following treatment, left eye
E11.37X 3	Type 2 diabetes mellitus with diabetic macular edema, resolved following treatment, bilateral
E11.37X 9	Type 2 diabetes mellitus with diabetic macular edema, resolved following treatment, unspecified eye
E11.39	Type 2 diabetes mellitus with other diabetic ophthalmic complication
E11.4	Type 2 diabetes mellitus with neurological complications
E11.40	Type 2 diabetes mellitus with diabetic neuropathy, unspecified
E11.41	Type 2 diabetes mellitus with diabetic mononeuropathy
E11.42	Type 2 diabetes mellitus with diabetic polyneuropathy
E11.43	Type 2 diabetes mellitus with diabetic autonomic (poly)neuropathy
E11.44	Type 2 diabetes mellitus with diabetic amyotrophy
E11.49	Type 2 diabetes mellitus with other diabetic neurological complication
E11.5	Type 2 diabetes mellitus with circulatory complications
E11.51	Type 2 diabetes mellitus with diabetic peripheral angiopathy without gangrene
E11.52	Type 2 diabetes mellitus with diabetic peripheral angiopathy with gangrene
E11.59	Type 2 diabetes mellitus with other circulatory complications
E11.6	Type 2 diabetes mellitus with other specified complications
E11.61	Type 2 diabetes mellitus with diabetic arthropathy
E11.610	Type 2 diabetes mellitus with diabetic neuropathic arthropathy
E11.618	Type 2 diabetes mellitus with other diabetic arthropathy
E11.62	Type 2 diabetes mellitus with skin complications
E11.620	Type 2 diabetes mellitus with diabetic dermatitis
E11.621	Type 2 diabetes mellitus with foot ulcer

© 2023 Benhammou JN et al. *JAMA Network Open.*

E11.622	Type 2 diabetes mellitus with other skin ulcer
E11.628	Type 2 diabetes mellitus with other skin complications
E11.63	Type 2 diabetes mellitus with oral complications
E11.630	Type 2 diabetes mellitus with periodontal disease
E11.638	Type 2 diabetes mellitus with other oral complications
E11.64	Type 2 diabetes mellitus with hypoglycemia
E11.640	Type 2 diabetes mellitus with hypoglycemia without coma
E11.641	Type 2 diabetes mellitus with hypoglycemia with coma
E11.649	Type 2 diabetes mellitus with hypoglycemia without coma
E11.65	Type 2 diabetes mellitus with hyperglycemia
E11.69	Type 2 diabetes mellitus with other specified complication
E11.8	Type 2 diabetes mellitus with unspecified complications
E11.9	Type 2 diabetes mellitus without complications
E13	Other specified diabetes mellitus
E13.0	Other specified diabetes mellitus with hyperosmolarity
E13.00	Other specified diabetes mellitus with hyperosmolarity without nonketotic hyperglycemic-hyperosmolar coma (NKHHC)
E13.01	Other specified diabetes mellitus with hyperosmolarity with coma
E13.1	Other specified diabetes mellitus with ketoacidosis
E13.10	Other specified diabetes mellitus with ketoacidosis without coma
E13.11	Other specified diabetes mellitus with ketoacidosis with coma
E13.2	Other specified diabetes mellitus with kidney complications
E13.21	Other specified diabetes mellitus with diabetic nephropathy
E13.22	Other specified diabetes mellitus with diabetic chronic kidney disease
E13.29	Other specified diabetes mellitus with other diabetic kidney complication
E13.3	Other specified diabetes mellitus with ophthalmic complications
E13.31	Other specified diabetes mellitus with unspecified diabetic retinopathy
E13.311	Other specified diabetes mellitus with unspecified diabetic retinopathy with macular edema
E13.319	Other specified diabetes mellitus with unspecified diabetic retinopathy without macular edema
E13.32	Other specified diabetes mellitus with mild nonproliferative diabetic retinopathy
E13.321	Other specified diabetes mellitus with mild nonproliferative diabetic retinopathy with macular edema
E13.3211	Other specified diabetes mellitus with mild nonproliferative diabetic retinopathy with macular edema, right eye
E13.3212	Other specified diabetes mellitus with mild nonproliferative diabetic retinopathy with macular edema, left eye
E13.3213	Other specified diabetes mellitus with mild nonproliferative diabetic retinopathy with macular edema, bilateral
E13.3219	Other specified diabetes mellitus with mild nonproliferative diabetic retinopathy with macular edema, unspecified eye
E13.329	Other specified diabetes mellitus with mild nonproliferative diabetic retinopathy without macular edema
E13.3291	Other specified diabetes mellitus with mild nonproliferative diabetic retinopathy without macular edema, right eye
E13.3292	Other specified diabetes mellitus with mild nonproliferative diabetic retinopathy without macular edema, left eye
E13.3293	Other specified diabetes mellitus with mild nonproliferative diabetic retinopathy without macular edema, bilateral
E13.3299	Other specified diabetes mellitus with mild nonproliferative diabetic retinopathy without macular edema, unspecified eye

© 2023 Benhammou JN et al. *JAMA Network Open.*

E13.33	Other specified diabetes mellitus with moderate nonproliferative diabetic retinopathy
E13.331	Other specified diabetes mellitus with moderate nonproliferative diabetic retinopathy with macular edema
E13.3311	Other specified diabetes mellitus with moderate nonproliferative diabetic retinopathy with macular edema, right eye
E13.3312	Other specified diabetes mellitus with moderate nonproliferative diabetic retinopathy with macular edema, left eye
E13.3313	Other specified diabetes mellitus with moderate nonproliferative diabetic retinopathy with macular edema, bilateral
E13.3319	Other specified diabetes mellitus with moderate nonproliferative diabetic retinopathy with macular edema, unspecified eye
E13.339	Other specified diabetes mellitus with moderate nonproliferative diabetic retinopathy without macular edema
E13.3391	Other specified diabetes mellitus with moderate nonproliferative diabetic retinopathy without macular edema, right eye
E13.3392	Other specified diabetes mellitus with moderate nonproliferative diabetic retinopathy without macular edema, left eye
E13.3393	Other specified diabetes mellitus with moderate nonproliferative diabetic retinopathy without macular edema, bilateral
E13.3399	Other specified diabetes mellitus with moderate nonproliferative diabetic retinopathy without macular edema, unspecified eye
E13.34	Other specified diabetes mellitus with severe nonproliferative diabetic retinopathy
E13.341	Other specified diabetes mellitus with severe nonproliferative diabetic retinopathy with macular edema
E13.3411	Other specified diabetes mellitus with severe nonproliferative diabetic retinopathy with macular edema, right eye
E13.3412	Other specified diabetes mellitus with severe nonproliferative diabetic retinopathy with macular edema, left eye
E13.3413	Other specified diabetes mellitus with severe nonproliferative diabetic retinopathy with macular edema, bilateral
E13.3419	Other specified diabetes mellitus with severe nonproliferative diabetic retinopathy with macular edema, unspecified eye
E13.349	Other specified diabetes mellitus with severe nonproliferative diabetic retinopathy without macular edema
E13.3491	Other specified diabetes mellitus with severe nonproliferative diabetic retinopathy without macular edema, right eye
E13.3492	Other specified diabetes mellitus with severe nonproliferative diabetic retinopathy without macular edema, left eye
E13.3493	Other specified diabetes mellitus with severe nonproliferative diabetic retinopathy without macular edema, bilateral
E13.3499	Other specified diabetes mellitus with severe nonproliferative diabetic retinopathy without macular edema, unspecified eye
E13.35	Other specified diabetes mellitus with proliferative diabetic retinopathy
E13.351	Other specified diabetes mellitus with proliferative diabetic retinopathy with macular edema
E13.3511	Other specified diabetes mellitus with proliferative diabetic retinopathy with macular edema, right eye
E13.3512	Other specified diabetes mellitus with proliferative diabetic retinopathy with macular edema, left eye
E13.3513	Other specified diabetes mellitus with proliferative diabetic retinopathy with macular edema, bilateral
E13.3519	Other specified diabetes mellitus with proliferative diabetic retinopathy with macular edema, unspecified eye

© 2023 Benhammou JN et al. *JAMA Network Open.*

E13.352	Other specified diabetes mellitus with proliferative diabetic retinopathy with traction retinal detachment involving the macula
E13.3521	Other specified diabetes mellitus with proliferative diabetic retinopathy with traction retinal detachment involving the macula, right eye
E13.3522	Other specified diabetes mellitus with proliferative diabetic retinopathy with traction retinal detachment involving the macula, left eye
E13.3523	Other specified diabetes mellitus with proliferative diabetic retinopathy with traction retinal detachment involving the macula, bilateral
E13.3529	Other specified diabetes mellitus with proliferative diabetic retinopathy with traction retinal detachment involving the macula, unspecified eye
E13.353	Other specified diabetes mellitus with proliferative diabetic retinopathy with traction retinal detachment not involving the macula
E13.3531	Other specified diabetes mellitus with proliferative diabetic retinopathy with traction retinal detachment not involving the macula, right eye
E13.3532	Other specified diabetes mellitus with proliferative diabetic retinopathy with traction retinal detachment not involving the macula, left eye
E13.3533	Other specified diabetes mellitus with proliferative diabetic retinopathy with traction retinal detachment not involving the macula, bilateral
E13.3539	Other specified diabetes mellitus with proliferative diabetic retinopathy with traction retinal detachment not involving the macula, unspecified eye
E13.354	Other specified diabetes mellitus with proliferative diabetic retinopathy with combined traction retinal detachment and rhegmatogenous retinal detachment
E13.3541	Other specified diabetes mellitus with proliferative diabetic retinopathy with combined traction retinal detachment and rhegmatogenous retinal detachment, right eye
E13.3542	Other specified diabetes mellitus with proliferative diabetic retinopathy with combined traction retinal detachment and rhegmatogenous retinal detachment, left eye
E13.3543	Other specified diabetes mellitus with proliferative diabetic retinopathy with combined traction retinal detachment and rhegmatogenous retinal detachment, bilateral
E13.3549	Other specified diabetes mellitus with proliferative diabetic retinopathy with combined traction retinal detachment and rhegmatogenous retinal detachment, unspecified eye
E13.355	Other specified diabetes mellitus with stable proliferative diabetic retinopathy
E13.3551	Other specified diabetes mellitus with stable proliferative diabetic retinopathy, right eye
E13.3552	Other specified diabetes mellitus with stable proliferative diabetic retinopathy, left eye
E13.3553	Other specified diabetes mellitus with stable proliferative diabetic retinopathy, bilateral
E13.3559	Other specified diabetes mellitus with stable proliferative diabetic retinopathy, unspecified eye
E13.359	Other specified diabetes mellitus with proliferative diabetic retinopathy without macular edema
E13.3591	Other specified diabetes mellitus with proliferative diabetic retinopathy without macular edema, right eye
E13.3592	Other specified diabetes mellitus with proliferative diabetic retinopathy without macular edema, left eye
E13.3593	Other specified diabetes mellitus with proliferative diabetic retinopathy without macular edema, bilateral
E13.3599	Other specified diabetes mellitus with proliferative diabetic retinopathy without macular edema, unspecified eye
E13.36	Other specified diabetes mellitus with diabetic cataract
E13.37	Other specified diabetes mellitus with diabetic macular edema, resolved following treatment
E13.37X1	Other specified diabetes mellitus with diabetic macular edema, resolved following treatment, right eye

© 2023 Benhammou JN et al. *JAMA Network Open.*

E13.37X2	Other specified diabetes mellitus with diabetic macular edema, resolved following treatment, left eye
E13.37X3	Other specified diabetes mellitus with diabetic macular edema, resolved following treatment, bilateral
E13.37X9	Other specified diabetes mellitus with diabetic macular edema, resolved following treatment, unspecified eye
E13.39	Other specified diabetes mellitus with other diabetic ophthalmic complication
E13.4	Other specified diabetes mellitus with neurological complications
E13.40	Other specified diabetes mellitus with diabetic neuropathy, unspecified
E13.41	Other specified diabetes mellitus with diabetic mononeuropathy
E13.42	Other specified diabetes mellitus with diabetic polyneuropathy
E13.43	Other specified diabetes mellitus with diabetic autonomic (poly)neuropathy
E13.44	Other specified diabetes mellitus with diabetic amyotrophy
E13.49	Other specified diabetes mellitus with other diabetic neurological complication
E13.5	Other specified diabetes mellitus with circulatory complications
E13.51	Other specified diabetes mellitus with diabetic peripheral angiopathy without gangrene
E13.52	Other specified diabetes mellitus with diabetic peripheral angiopathy with gangrene
E13.59	Other specified diabetes mellitus with other circulatory complications
E13.6	Other specified diabetes mellitus with other specified complications
E13.61	Other specified diabetes mellitus with diabetic arthropathy
E13.610	Other specified diabetes mellitus with diabetic neuropathic arthropathy
E13.618	Other specified diabetes mellitus with other diabetic arthropathy
E13.62	Other specified diabetes mellitus with skin complications
E13.620	Other specified diabetes mellitus with diabetic dermatitis
E13.621	Other specified diabetes mellitus with foot ulcer
E13.622	Other specified diabetes mellitus with other skin ulcer
E13.628	Other specified diabetes mellitus with other skin complications
E13.63	Other specified diabetes mellitus with oral complications
E13.630	Other specified diabetes mellitus with periodontal disease
E13.638	Other specified diabetes mellitus with other oral complications
E13.64	Other specified diabetes mellitus with hypoglycemia
E13.640	Other specified diabetes mellitus with hypoglycemia without coma
E13.641	Other specified diabetes mellitus with hypoglycemia with coma
E13.649	Other specified diabetes mellitus with hypoglycemia without coma
E13.65	Other specified diabetes mellitus with hyperglycemia
E13.69	Other specified diabetes mellitus with other specified complication
E13.8	Other specified diabetes mellitus with unspecified complications
E13.9	Other specified diabetes mellitus without complications
N25.1	Nephrogenic diabetes insipidus
O24	Diabetes mellitus in pregnancy, childbirth, and the puerperium
O24.1	Pre-existing type 2 diabetes mellitus, in pregnancy, childbirth and the puerperium
O24.11	Pre-existing type 2 diabetes mellitus, in pregnancy
O24.111	Pre-existing type 2 diabetes mellitus, in pregnancy, first trimester
O24.112	Pre-existing type 2 diabetes mellitus, in pregnancy, second trimester
O24.113	Pre-existing type 2 diabetes mellitus, in pregnancy, third trimester
O24.119	Pre-existing type 2 diabetes mellitus, in pregnancy, unspecified trimester
O24.12	Pre-existing type 2 diabetes mellitus, in childbirth

© 2023 Benhammou JN et al. *JAMA Network Open.*

O24.13	Pre-existing type 2 diabetes mellitus, in the puerperium
O24.3	Unspecified pre-existing diabetes mellitus in pregnancy, childbirth and the puerperium
O24.31	Unspecified pre-existing diabetes mellitus in pregnancy
O24.311	Unspecified pre-existing diabetes mellitus in pregnancy, first trimester
O24.312	Unspecified pre-existing diabetes mellitus in pregnancy, second trimester
O24.313	Unspecified pre-existing diabetes mellitus in pregnancy, third trimester
O24.319	Unspecified pre-existing diabetes mellitus in pregnancy, unspecified trimester
O24.32	Unspecified pre-existing diabetes mellitus in childbirth
O24.33	Unspecified pre-existing diabetes mellitus in the puerperium
R73.03	Prediabetes
Z13.1	Encounter for screening for diabetes mellitus
Z83.3	Family history of diabetes mellitus
Z86.32	Personal history of gestational diabetes

eTable 1i- Hypertension ICD 9/10 codes

ICD 9/10	CONCEPT_NAME
401	essential hypertension
401.1	benign hypertension
401.9	unspecified essential hypertension

eTable 1j- Hepatic encephalopathy ICD 9/10 codes

ICD 9/10	CONCEPT_NAME
572.2	Hepatic encephalopathy
B16.2	Acute hepatitis B without delta-agent with hepatic coma
B17.11	Acute hepatitis C with hepatic coma
B19.0	Unspecified viral hepatitis with hepatic coma
B19.11	Unspecified viral hepatitis B with hepatic coma
B19.21	Unspecified viral hepatitis C with hepatic coma
K70.41	Alcoholic hepatic failure with coma
K72.11	Chronic hepatic failure with coma
K72.91	Hepatic failure, unspecified with coma

eTable 1k- Hepatorenal syndrome ICD 9/10 codes

ICD 9/10	CONCEPT_NAME
572.4	Hepatorenal syndrome

eTable 1l- Ascites ICD 9/10 codes

ICD 9/10	CONCEPT_NAME
789.5	Ascites
789.59	Other ascites
K65.2	Spontaneous bacterial peritonitis
K70.11	Alcoholic hepatitis with ascites
K70.31	Alcoholic cirrhosis of liver with ascites

© 2023 Benhammou JN et al. *JAMA Network Open.*

R18.8	Other ascites

eTable 1m- Spontaneous bacterial peritonitis ICD 9/10 codes

ICD 9/10	CONCEPT_NAME
567.23	spontaneous bacterial peritonitis
K65.2	spontaneous bacterial peritonitis

eTable 1n- Bleeding esophageal and gastric varices ICD 9/10 codes

ICD 9/10	CONCEPT_NAME
456, 456.2	Esophageal varices with bleeding
I85.01	Esophageal varices with bleeding
I86.41	Gastric varices with bleeding
I85.11	Secondary esophageal varices with bleeding

eTable 1o- Liver transplantation ICD 9/10 codes

ICD 9/10	CONCEPT_NAME
V42.7	Liver transplant status
Z94.4	Liver transplant status

© 2023 Benhammou JN et al. *JAMA Network Open.*

eTable 2- Human immunodeficiency virus (HIV), hepatitis C, hepatitis B, AST, ALT and platelet LOINCs

Test	LOINC
HIV 1 Ab + HIV 2 Ab	56888-1
HIV 1 RNA	62469-2
HIV RNA	20447-9
HIV RNA	29541-0
HIV RNA	62469-2
HIV RNA	25835-0
HIV 1 + 2 Ab	31201-7
HIV 1 + HIV 2 Ab & HIV 1 p24Ag	75666-8
HIV 1 Ab + HIV 2 Ab	80387-4
HIV 1 Ab	29893-5
HIV 1 Ab & HIV 2 Ab	80203-3
HIV 1 Ab & HIV 2 Ab	68961-2
	86233-4
HIV 1 Ab & HIV 2 Ab	69668-2
HIV 1 Ab	5221-7
HIV 1 (presence)	32571-2
Anti-HIV 1 + Anti-HIV 2	49580-4
HIV 1 Ab + HIV 2 Ab	31201-7
HIV 1 Ab + HIV 2 Ab	80387-4
Hepatitis C virus RNA	3014759
Hepatitis C virus RNA	3016770
Hepatitis C virus RNA	3018447
Hepatitis C virus RNA	3021125
Hepatitis C virus RNA	3024429
Hepatitis C virus RNA	3033192
Hepatitis C virus RNA	3052023
Hepatitis B virus surface Ag in serum or plasma by immunoassay	3019510
Hepatitis B virus surface Ag in serum or plasma by neutralization test	3025267
AST	545004-4
AST	1916-6
AST	16325-3
AST	27344-1
AST	1920-8
AST	2325-9
AST	44786-2
AST	30239-8
AST	88112-8
AST	100739-2
AST	48136-6
ALT	77144-4
ALT	16325-3
ALT	1916-6
ALT	1742-6
ALT	100738-4

© 2023 Benhammou JN et al. *JAMA Network Open.*

ALT	48134-1
ALT	1743-4
ALT	1744-2
ALT	44785-4
Platelets.reticulated/100 platelets	51633-6
Platelets.reticulated/100 platelets	71693-6
Platelets.large/Platelets	97994-8
Platelets.large/Platelets	48386-7
Platelets.reticulated/platelets.total	70032-8
Platelets.agranular	33216-3
Platelets.reticulated	51632-8
Platelets.large fragments	15201-7
Platelets.giant	5908-9
Platelets.large	32146-3
Platelets.small	32208-1
Bizarre platelets	60455-3
Platelets.large	34167-7
Platelets.reticulated	71692-8
Platelets	12243-2
Platelets.large	96354-6
Platelets	26515-7
Platelets	26516-5
Platelets	79427-1
Platelets	49497-1
Platelets	778-1
Platelets	5907-1
Platelets	777-3
Platelets	13056-7
Platelets	74775-8
Platelets	97995-5
Platelets	74464-9
Platelets.giant/100 leukocytes	32712-2
Platelets	9317-9
Platelet genotype	34977-9
Platelet distribution width	51631-0
Platelet morphology finding	11125-2
Platelet mass	55206-7
Platelet mean volume	28542-9
Platelet anisocytosis	51640-1
Platelet clump	7796-6
Platelet mean diameter	30459-2
Platelet clump	40741-1
Platelet component distribution width	76137-9
Platelet mean diameter	775-7
Platelet mean volume	776-5
Platelet satellitism	18312-9
Mean platelet component	76683-2

© 2023 Benhammou JN et al. *JAMA Network Open.*

Platelet distribution width	32207-3
Platelet dry mass distribution width	76685-7
Mean platelet dry mass	76684-0
Platelet dense bodies	79768-8
Platelet mean volume	32623-1
Platelet mean volume	32711-4

© 2023 Benhammou JN et al. *JAMA Network Open.*

eTable 3- ICD 9 codes for other gastrointestinal malignancies

Site	ICD9	Description
**Colon**	153	Malignant neoplasm of colon
Hepatic flexure	153.0	
Transverse colon	153.1	
Descending colon	153.2	Left colon
Sigmoid colon	153.3	Sigmoid (flexure)
Cecum	153.4	Ileocecal valve
Appendix	153.5	
Ascending colon	153.6	Right colon
Splenic flexure	153.7	
Other specified sites of large intestine	153.8	Malignant neoplasm of contiguous or overlapping sites of colon whose point of origin cannot be determined
Colon, unspecified	153.9	Large intestine NOS
**Rectum**	154	Malignant neoplasm of rectum, rectosigmoid junction, and anus
Rectosigmoid junction	154.0	Colon with rectum/Rectosigmoid (colon)
Rectum	154.1	Rectal ampulla
Anal canal	154.2	Anal spincter
Anus, unspecified	154.3	
Other	154.8	Anorectum/Cloacogenic zone/Malignant neoplasm of contiguous or overlapping sites of rectum, rectosigmoid junction, and anus whose point of origin cannot be determined
**Stomach**	151	Malignant neoplasm of stomach
Cardia	151.0	Cardiac orifice/Cardio-esophageal junction
Pylorus	151.0	Prepylorus/Pyloric canal
Pyloric antrum	151.2	Antrum of stomach NOS
Fundus of stomach	151.3	
Body of stomach	151.4	
Lesser curvature, unspecified	151.5	Lesser curvature, not classifiable to 151.1-151.4
Greater curvature, unspecified	151.6	Greater curvature, not classifiable to 151.0-151.4
Other specified sites of stomach	151.8	Anterior wall, not classifiable to 151.0-151.4/Posterior wall, not classifiable to 151.0-151.4/Malignant neoplasm of contiguous or overlapping sites of stomach whose point of origin cannot be determined
Stomach, unspecified	151.9	Carcinoma ventriculi/Gastric cancer
**Pancreas**	157	Malignant neoplasm of pancreas
Head of pancreas	157.0	
Body of pancreas	157.1	
Tail of pancreas	157.2	
Pancreatic duct	157.3	Duct of: Santorini/Wirsung
Islets of Langerhans	157.4	Islets of Langerhans, any part of pancreas/Use additional code to identify any functional activity

© 2023 Benhammou JN et al. *JAMA Network Open.*

Other specified sites of pancreas	157.8	Ectopic pancreatic tissue/Malignant neoplasm of contiguous or overlapping sites of pancreas whose point of origin cannot be determined
Pancreas, part unspecified	157.9	

© 2023 Benhammou JN et al. *JAMA Network Open*.

eTable 4- Association between AO and other risk factors for HCC using APRI score to identify HCC cases.

**Characteristic**	**Non-cirrhosis (n = 263,148)**		**Cirrhosis (n = 33,355)**	
	**Multivariable aHR (95% CI)**	***P*-value**	**Multivariable aHR (95% CI)**	***P*-value**
Agent Orange	1.001 (0.853-1.175)	0.9893	0.8142 (0.923-1.107)	0.8142
Age (at deployment)	1.017 (0.997-1.037)	0.0937	**0.971 (0.953-0.990)**	**0.0030**
Hispanic or Latino**	0.967 (0.697-1.343)	0.8423	**1.503 (1.297-1.742)**	**<0.0001**
Non-Hispanic Black	1.132 (0.924-1.387)	0.2317	**1.165 (1.040-1.304)**	**0.0081**
Smoking	1.286 (0.975-1.698)	0.0753	1.086 (0.887-1.328)	0.4247
Obesity	1.004 (0.786-1.282)	0.9764	0.941 (0.812-1.090)	0.4150
Viral hepatitis	**2.459 (1.080-2.906)**	**<0.0001**	**3.715 (3.255-4.241)**	**<0.0001**
Alcohol liver disease	1.063 (0.906-1.249)	0.4530	**1.319 (1.187-1.466)**	**<0.0001**
NASH/NAFLD[#]	**2.418 (1.954-2.993)**	**<0.0001**	**1.898 (1.699-2.121)**	**<0.0001**
HIV	1.103 (0.933-1.304)	0.2530	0.974 (0.888-1.067)	0.5707
Autoimmune hepatitis[##]	1.811 (0.978-3.352)	0.0588	1.121 (0.866-1.452)	0.3842
Hemochromatosis	**1.911 (1.174-3.110)**	**0.0092**	**1.306 (1.060-1.609)**	**0.0122**
Alpha-1-antri trypsin	**1.658 (0.969-1.838)**	**0.0652**	1.134 (0.944-1.363)	0.1792
Secondary or unspecific biliary cirrhosis[&]	-	-	1.288 (0.906-1.831)	0.1587
Dyslipidemia	**0.477 (0.379-0.600)**	**<0.0001**	**0.455 (0.405-0.510)**	**<0.0001**
Hypertension	**1.668 (1.270-2.190)**	**0.0002**	1.163 (0.995-1.360)	0.0572
Diabetes[&&]	**1.448 (1.085-1.933)**	**0.0120**	1.076 (0.915-1.267)	0.3749
Diabetes and Obesity interactions	1.009 (0.715-1.424)	0.9591	0.956 (0.785-1.164)	0.6519

*adjusting for age at deployment, race and ethnicity; **White individuals used as reference group; [#]combined NAFLD and NASH in multivariable model; [##]combined HCV and HBV; [&]no cases in the non-cirrhosis group; [&&]obesity and diabetes interactions assessed which was not significant in multivariable model.

Abbreviations: aHR=adjusted hazard ratio; NAFLD=nonalcoholic fatty liver disease; NASH=nonalcoholic steatohepatitis; HIV=human immunodeficiency virus.

© 2023 Benhammou JN et al. *JAMA Network Open.*

eMethods:

AO exposure is not associated with incident HCC with age stratification

To further explore the finding of age at deployment being inversely associated with incident HCC, we assessed the interactions between AO exposure and age. Independently of cirrhosis status, there was no significant association between AO exposure and age after adjusting for race and ethnicity. Similarly, early age (decade 20 in years) was not significantly associated with HCC in the cirrhosis (aHR = 1.00, 95% CI 0.92-1.09, p=0.99) and non-cirrhosis (aHR = 0.96, 95% CI 0.81-1.14, p=0.65) groups (**eTable 5**).

Association between smoking and alcohol with incident HCC

In the non-cirrhosis group, age significantly interreacted with smoking (aHR = 1.78, 95% CI 1.30-1.08, p=0.0473), after adjusting for AO exposure, race and ethnicity. After stratifying age into decades (20-60), we assessed the association of each decade with incident HCC. We found that young patients (decade = 20) had a significantly higher risk for incident HCC in both cirrhosis (aHR= 1.32, 95% CI 1.01-1.63, p=0.0078) and cirrhosis groups (aHR = 1.91, 95% CI 1.38-2.66, p=0.0001), compared to other decades (older decades of 30-60) (e**Table 6**)

No significant interactions were identified between age and alcohol, however after stratifying age by decade we found that young age was significantly associated with incident HCC. Veterans in their 20s (aHR = 2.05, 95% CI 1.84-2.28, p<0.0001) and 30s (aHR = 1.89, 95% CI 1.27-2.82, p=0.0016), compared to older Veterans (decades 40-60) (**eTable 7**).

© 2023 Benhammou JN et al. *JAMA Network Open.*

eTable 5- Association between AO and other risk factors for HCC using inverse probability weighing

	**Multivariable aHR (95% CI)**	***P*-value**
Agent Orange	0.899 (0.804-1.006)	0.0637
**Cirrhosis**	**6.106 (5.494-6.785)**	**<0.0001**
Age (at deployment)	0.989 (0.972-1.007)	0.2238
Hispanic or Latino	1.194 (0.974-1.463)	0.0877
Non-Hispanic Black	1.106 (0.961-1.273)	0.1592
Smoking	1.146 (0.901-1.457)	0.2663
Obesity	0.987 (0.829-1.176)	0.8873
**Viral hepatitis**	**3.203 (2.842-3.611)**	**<0.0001**
**Alcohol liver disease**	**1.286 (1.151-1.436)**	**<0.0001**
**NASH/NAFLD[#]**	**2.291 (2.008-2.615)**	**<0.0001**
HIV	0.977 (0.868-1.100)	0.7049
Autoimmune hepatitis[##]	1.024 (0.700-1.497)	0.9031
**Hemochromatosis**	**2.512 (1.924-3.280)**	**<0.0001**
Alpha-1-antri trypsin	1.298 (0.965-1.746)	0.0847
Secondary or unspecific biliary cirrhosis[&]	0.780 (0.078-7.821)	0.8330
**Dyslipidemia**	**0.439 (0.370-0.521)**	**<0.0001**
**Hypertension**	**1.337 (1.119-1.598)**	**0.0014**
**Diabetes[&&]**	**1.250 (1.017-1.537)**	**0.0343**
Diabetes and Obesity interactions	1.134 (0.888-1.449)	0.3139

© 2023 Benhammou JN et al. *JAMA Network Open.*

eTable 6- Age (in years) stratification for AO exposure.

	Cirrhosis (n = 35,877)				Non-cirrhosis (n = 260,628)		
**Age by decade**	**HR**	**95% CI**	**P-value**	**Age by decade**	**HR**	**95% CI**	**P-value**
age=20	1.009	0.922-1.103	0.8532	age=20	0.979	0.825-1.162	0.8072
age=30	0.874	0.585-1.304	0.5086	age=30	0.818	0.554-1.210	0.3151
age=40	0.757	0.337-1.698	0.4989	age=40	0.684	0.306-1.531	0.3555
age=50	0.656	0.194-2.217	0.4969	age=50	0.572	0.167-1.959	0.3736
age=60	0.568	0.111-2.898	0.4961	age=60	0.478	0.091-2.514	0.3835

eTable 7- Age (in years) stratification for tobacco use.

	Cirrhosis (n = 35,877)				Non-cirrhosis (n = 260,628)		
**Age by decade**	**HR**	**95% CI**	**P-value**	**Age by decade**	**HR**	**95% CI**	**P-value**
**age=20**	**1.324**	**1.077-1.627**	**0.0078**	**age=20**	**1.915**	**1.380-2.659**	**0.0001**
age=30	0.869	0.460-1.643	0.6665	age=30	1.110	0.698-1.765	0.6589
age=40	0.571	0.154-2.120	0.4024	age=40	0.643	0.249-1.664	0.3629
age=50	0.375	0.051-2.763	0.3358	age=50	0.373	0.085-1.628	0.1895
age=60	0.246	0.017-3.610	0.3064	age=60	0.216	0.029-1.606	0.1343

eTable 8- Age (in years) stratification for alcohol use.

	Cirrhosis (n = 35,877)				Non-cirrhosis (n = 260,628)		
**Age by decade**	**HR**	**95% CI**	**P-value**	**Age by decade**	**HR**	**95% CI**	**P-value**
**age=20**	**2.048**	**1.843-2.276**	**<.0001**	**age=20**	**1.2658**	**1.0701-1.4973**	**0.0059**
**age=30**	**1.894**	**1.272-2.819**	**0.0016**	**age=30**	0.9997	0.633-1.5789	0.9990
age=40	1.751	0.782-3.922	0.1730	age=40	0.7895	0.3098-2.0123	0.6206
age=50	1.620	0.479-5.480	0.4383	age=50	0.6235	0.1501-2.5898	0.5156
age=60	1.498	0.293-7.666	0.6279	age=60	0.4924	0.0726-3.3408	0.4683

© 2023 Benhammou JN et al. *JAMA Network Open.*
