# Supplementary material for: Exposure to Agent Orange and Hepatocellular Carcinoma Among US Military Personnel
Source: JAMA Netw Open. 2023 Dec 4;6(12):e2346380. doi: 10.1001/jamanetworkopen.2023.46380 (PMC10696483; doi:10.1001/jamanetworkopen.2023.46380)
Supplement: Supplement 2. — Data Sharing Statement [file jamanetwopen-e2346380-s002.pdf]

Data Sharing Statement

Data
Data available: Yes
Data types: Data dictionary
How to access data: All data dictionaries are listed in supplemental tables. Corresponding author can also be reached with further questions at jbenhammou@mednet.ucla.edu. We are not able to provide patient-level data given real social security numbers were used and all data must remain behind the VA firewall as approved by the local IRB.
When available: With publication

Supporting Documents
Document types: None

Additional Information
Who can access the data: Codes will be made available to anyone requesting the data who have Veterans Affairs approvals for VA studies.
Types of analyses: Codes used to identify the cohort can be shared with VA or VA affiliated investigators who have the appropriate approvals for the study. ICDs codes used for the study are all available in the supplemental data.
Mechanisms of data availability: These are available in supplemental figure or can corresponding author can be reached for further questions.
